# The Diagnostic Value of the FIB-4 Index for Staging Hepatitis B-Related Fibrosis: A Meta-Analysis

**DOI:** 10.1371/journal.pone.0105728

**Published:** 2014-08-28

**Authors:** Yuanyuan Li, Yu Chen, Ying Zhao

**Affiliations:** Department of Laboratory Medicine, The First Affiliated Hospital of the Medical College, Zhejiang University, Hangzhou, China; University of Utah School of Medicine, United States of America

## Abstract

**Background:**

Liver fibrosis stage is an important factor in determining prognosis and need for treatment in patients infected with hepatitis B virus (HBV). Liver biopsies are typically used to assess liver fibrosis; however, noninvasive alternatives such as the FIB-4 index have also been developed.

**Aims:**

To quantify the accuracy of the FIB-4 index in the diagnosis of HBV related fibrosis and cirrhosis.

**Methods:**

A meta-analysis of studies comparing the diagnostic accuracy of the FIB-4 index vs. liver biopsy in HBV-infected patients was performed using studies retrieved from the following databases: PubMed, Ovid, EMBASE, the Cochrane Library, the Chinese National Knowledge Infrastructure and the Chinese Biology Medicine disc. A hierarchical summary receiver operating curves model and bivariate model were used to produce summary receiver operating characteristic curves and pooled estimates of sensitivity and specificity. The heterogeneity was explored with meta-regression analysis. Publication bias was detected using Egger’s test and the trim and fill method.

**Results:**

12 studies (*N* = 1,908) and 10 studies (*N* = 2,105) were included in the meta-analysis for significant fibrosis and cirrhosis, respectively. For significant fibrosis, the area under the hierarchical summary receiver operating curve (AUHSROC) was 0.78 (95% CI = 0.74–0.81). The recommended cutoff value was between 1.45 and 1.62, and the AUHSROC, summary sensitivity and specificity were 0.78 (95% CI = 0.74–0.81), 0.65 (95% CI = 0.56–0.73) and 0.77 (95% CI = 0.7–0.83), respectively. For cirrhosis, the AUHSROC was 0.89 (95% CI = 0.85–0.91). The recommended cutoff value was between 2.9 and 3.6, and the AUHSROC, summary sensitivity and specificity were 0.96 (95% CI = 0.92–1.00), 0.42 (95% CI = 0.36–0.48) and 0.96 (95% CI = 0.95–0.97), respectively. No publication bias was detected.

**Conclusions:**

The FIB-4 index is valuable for detecting significant fibrosis and cirrhosis in HBV-infected patients, but has suboptimal accuracy in excluding fibrosis and cirrhosis.

## Introduction

An accurate assessment of liver fibrosis in patients with hepatitis virus B (HBV) infection is essential not only in determining whether and when to initiate antiviral therapy, but also in predicting long-term clinical prognosis [1]–[3]. For example, with regard to antiviral therapy, it is known that maintenance of viral suppression can reduce liver-related complications in chronic hepatitis B (CHB) patients [1]–[3]. Furthermore, assessing prognosis in patients with cirrhosis is required to closely follow the potential development of hepatocellular carcinoma and other complications [2], [4].

To date, liver biopsy remains the gold standard for assessing liver fibrosis; however, it does have some limitations. The invasive nature of the biopsy is associated with patient discomfort, and can cause rare but important complications [5]. Furthermore, its accuracy is affected by sampling error and variability in pathological interpretation [6], [7], and the dynamic process of liver fibrosis related to disease progression and regression cannot be easily quantified. An ideal diagnostic index should be accurate, noninvasive, inexpensive, convenient and readily available. The limitations of the liver biopsy have lead many clinicians to develop noninvasive indexes, and most attention has been focused on whether noninvasive indexes can detect the presence or absence of significant fibrosis (i.e., ≥F2), severe fibrosis (i.e., ≥F3) and cirrhosis (i.e., ≥F4) according to the METAVIR histological score [8].

Currently, there are several categories of non-invasive indexes. Measures of hyaluronic acid, collagen, laminin and YKL-40 are direct laboratory indexes, but these are usually not routinely available. Indirect laboratory indexes are calculated from routine laboratory data, and include the aspartate aminotransferase (AST) to alanine aminotransferase (ALT) ratio (AAR), the AST to platelet (PLT) ratio index (APRI), the cirrhosis discriminant score (CDS), the age-PLT index (API), the FIB-4 index (see below), Lok’s model and the red cell distribution width (RDW) to platelet ratio [9]–[11]. While some of the calculations for these indexes are simple and accessible, some are more complex [11]. Assessment of these indexes has been reviewed and found to vary from bad to excellent [9], [11]; however, relevant systematic reviews in the context of HBV are rare. Thus, no current index has satisfied all the standards of the ideal diagnostic index [12].

The FIB-4 index is calculated using the formula: FIB-4 = Age (years)×AST (U/L)/[PLT(10^9^/L)×ALT^1/2^ (U/L)]. The theoretical basis for this index has been previously described [13], and adheres to the following logic: (1) age is considered to be relevant to disease duration and is associated with more severe fibrosis; (2) elevations in AST more than ALT has been related to both delayed clearance of AST relative to ALT, and to the mitochondrial injury associated with more advanced fibrosis; (3) thrombocytopenia has been associated with the progression of fibrosis and worsening portal hypertension that not only destroys platelets by sequestration in the enlarged spleen, but also decreases the production of thrombopoietin by hepatocytes. Based on these foundations, the FIB-4 index was first applied to assess hepatic fibrosis in the context of human immunodeficiency virus (HIV) and hepatitis C virus (HCV) infection [14], [15].

The diagnostic value of the FIB-4 index is attractive because measures of AST, ALT and PLT are routine and inexpensive tests in the clinical laboratory, and the calculation is simple. Numerous studies have assessed the diagnostic performance of the FIB-4 index in HBV-related fibrosis and cirrhosis [10], [11], [13], [16]–[32], with several showing that the FIB-4 index was superior in comparison to other non-invasive indexes [9], [13], [16]. Despite the benefit shown by these studies, the utility of the FIB-4 index remains controversial. Thus, the aim of the current study was to perform a meta-analysis of diagnostic tests for predicting the accuracy of the FIB-4 index in predicting significant fibrosis (F2–F4 vs. F0–F1), severe fibrosis (F3–F4 vs. F0–F2) and cirrhosis (F4 vs. F0–F3) in patients with HBV infection.

## Materials and Methods

### Ethics statement

The data of this meta-analysis was extracted from published studies. So the data were analyzed anonymously.

### Literature and search strategy

The following databases were searched without the use of time limitations: PubMed, Ovid, EMBASE, the Cochrane Library, the Chinese National Knowledge Infrastructure (CNKI) and the Chinese Biology Medicine disc (CBMdisc). The search strategy to identify all relevant articles involved the use of the following key words: FIB-4, aspartate aminotransferase, AST, alanine aminotransferase, ALT, platelet, PLT, hepatitis B, fibrosis and cirrhosis. For example, File S1 and S2 displayed the search strategy of Ovid and PubMed respectively. Additional studies were identified via a manual review of the reference lists of identified studies and review articles. This literature search was performed in November 2013.

### Inclusion criteria

Studies were deemed eligible if they met the following inclusion criteria: 1) the study evaluated the performance of the FIB-4 index for the diagnosis of fibrosis in mono-HBV-infected patients before antiviral therapy. Studies including patients with other causes of liver disease were included if data of HBV-infected patients could be extracted. 2) Liver biopsy was used as the reference standard for assessing fibrosis. METAVIR [8] or comparable staging systems (i.e., Batts and Ludwig [33], Scheuer [34] or Ishak [35]) were applied to stage fibrosis. Significant fibrosis was defined as F≥2 for METAVIR, Batts and Ludwig, and Scheuer staging systems; or F≥3 for the Ishak system. Severe fibrosis was defined as F≥3 for METAVIR, Batts and Ludwig, and Scheuer staging systems. Cirrhosis was defined as F≥4 for METAVIR, Batts and Ludwig, and Scheuer staging systems; or F≥5 for the Ishak system. 3) Data could be extracted to allow the construction of at least one 2×2 table of test performance. 4) The study included more than 40 patients; otherwise it was excluded because of low statistical power and poor reliability.

### Quality assessment and data extraction

Two reviewers (Drs. Li and Zhao) independently evaluated the eligibility of each study according to the inclusion criteria described above, and assessed methodological quality according to the Quality Assessment of Diagnostic Accuracy Studies-2 (QUADAS-2) tool [36]. Discrepancies were resolved by consensus agreement. Note – some specific issues were defined before assessment, e.g. CHB was defined as hepatitis B surface antigen positive for more than 6 months. With regard to disease progression bias, the time interval between the determination of the FIB-4 index and liver biopsy was no longer than 7 days. As the FIB-4 index was calculated from four objective measures (ALT, AST, PLT and age), the item relating to blinding of the test interpreter to results of the reference standard was omitted. In addition to 2×2 tables of test performance, two kinds of data (patient related data and study related data) were also extracted. The patient related data included mean age, gender distribution, region and prevalence of the fibrosis stages. The study related data included sample size, interval time between determination of the FIB-4 index and liver biopsy, the size of liver biopsy, histological scoring system, blinded interpretation of the biopsy, and the cutoff value of the FIB-4. To avoid double counting of data, when multiple pairs of sensitivity or specificity were reported in one study, we consistently used the data with the highest Youden index (sensitivity + specificity-1) for meta-analysis [37], except for subgroup analysis based on different cutoff values.

### Statistical analyses

For meta-analyses, a bivariate random effects model [38] was used to calculate summary estimates of sensitivity, specificity, positive likelihood ratio (PLR) and negative likelihood ratio (NLR), and to fit a hierarchical summary receiver-operating characteristic (HSROC) curve [39]. These models take into account potential threshold effects and the correlation between sensitivity and specificity. They also allow addition of covariates for investigation of potential sources of heterogeneity, thus are standard methods recommended for meta-analyses of diagnostic tests [40], [41]. Additionally, the following guidelines have been suggested for interpretation of the area under the hierarchical summary receiver-operating characteristic curve (AUHSROC): an area of 1.0 indicates perfect discrimination, and 0.90 to 1.0 has been classified as excellent, 0.80 to less than 0.90 as good, 0.70 to less than 0.80 as fair, and less than 0.70 as poor [42], [43].

Multiple sources of heterogeneity frequently exist in diagnostic studies. In addition to visual assessment with the use of the forest plots, we formally quantified the extent of heterogeneity by calculating the inconsistency index (*I^2^* statistics) [44]. Statistically significant heterogeneity was considered present at *I^2^*>50%. To explore the source of heterogeneity, meta-regression and subgroup analysis were performed. The potential factors evaluated by meta-regression analysis were mean age of subjects, prevalence of fibrosis stages, disease spectrum, a consecutive or random sample enrollment, interval between FIB-4 index determination and liver biopsy, the liver blinded biopsy interpretation and a predefined cutoff value.

With respect to publication bias, the funnel plot is a basic and routine method for detecting biases, but it is subjective and qualitative. To counter these limitations, several quantitative methods such as Egger’s test [45] and the trim and fill method [46] have been developed. Egger’s test quantifies the degree of funnel plot asymmetry as measured by the intercept from regression of standard normal deviates against precision, but its capacity to detect bias is limited when meta-analyses are based on a limited number of small trials [45]. The trim and fill method is a nonparametric method for estimating the number of missing studies that might exist in a meta-analysis and the effect that these studies might have had on its outcome. This method also provides effective and relatively powerful tests for evaluating the existence of such publication bias [46]. To be cautious, the publication bias was assessed with two distinct methods. Statistical analyses were conducted using Review Manager 5.2 (The Cochrane Collaboration) and STATA 11.0 (Stata Corp., College Station, TX, USA), notably with the user-written ‘midas’ and ‘metandi’ programs for STATA.

## Results

### Search results

**Figure 1:**
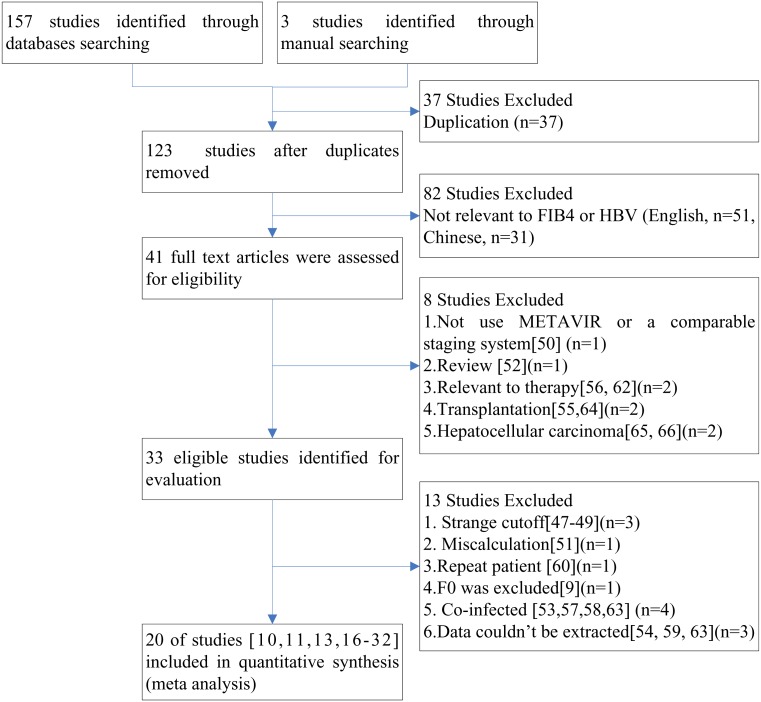
Flow chart of article selection.

One hundred and sixty studies were retrieved based on the described search strategies. One hundred and forty studies [9], [47]–[66] were excluded in accord with our exclusion criteria (Figure 1). Xu et al. defined cutoff values for the FIB-4 index as 223.70 for predicting significant fibrosis and 808.77 for cirrhosis [47], and Gumusay et al. reported that the average FIB-4 index for healthy controls was 11.7±5.8 [48]. These cutoff values were much larger than that of other studies, which ranged from 0.8 to 4.9, and we could not calculate similar cutoff values from the data included in these articles, thus they were excluded [47], [48]. Wong et al. calculated cutoff values based on a >90% sensitivity to exclude and >90% specificity to confirm advanced liver fibrosis [49]. This method was different from other studies, so it was also excluded. Two studies [13], [60] included the same patient populations, thus the study with the smaller sample size and data that could not be extracted was excluded [60]. As the true negative and false positive patients of the study by Jing et al. were underestimated because they excluded non-fibrotic samples (i.e.,F0), we excluded it [9]. Ultimately, 20 studies [10], [11], [13], [16]–[32] were eligible for evaluation, and their characteristics are listed in Table 1. Although two studies [22], [32] were written by the same author, the patients were collected at different times and the study with the smaller sample size [32] was excluded for further sensitivity analysis.

**Table 1:** Characteristics of studies eligible for meta-analysis.

Author, Year, Region	Number(male%)	Age(years)	IntervalBetweenBiopsyand FIB-4	Liver BiopsyScoringsystem	LiverBiopsyLength	Blinded
Chen[10], 2013, China	148 (66%)	40.36±11.2	≤7d	Metavir	>15 mm	Yes
Kim[13], 2010, Korea	668 (66%)	39.1±14.8	≤2d	Batts and Ludwig	>15 mm	Yes
Zhu[29], 2012, China	159 (71%)	42 (18∼62)	unclear	METAVIR	≥15 mm	unclear
Ucar[18], 2013, Turkey	73 (64%)	42.81±12.86	unclear	METAVIR	unclear	Yes
Gong[21], 2013, China	41 (73%)	50.8±10.3	unclear	METAVIR	unclear	unclear
Wang[20], 2013, China	231 (68%)	34.1±9.8	<1d	Scheuer	>15 mm	Yes
Ji[17], 2011, China	313 (69%)	35.6±11.2	1d	METAVIR	20 mm	unclear
Başar[25], 2013, Turkey	76 (55%)	unclear	<1d	METAVIR	>10 mm	Yes
Bonnard[19], 2010, France	59 (68%)	35±9	0.5–10 m	METAVIR	21±6 mm	Yes
Erdogan[11], 2013, Turkey	221 (63%)	43.68±12.56	≤1d	Ishak	unclear	Yes
Wu[30], 2010, China	78 (85%)	32.6±12.3	unclear	METAVIR	>15 mm	unclear
Mallet[16], 2009, France	138 (71%)	42±15	<1d	METAVIR	17.6±6.8	unclear
Seto[24], 2011, China	237 (68%)	38.2 (18∼63)	same time	Ishak	≥15 mm	Yes
Zhu[27], 2011, China	175 (78%)	36.5±9.4	≤7d	METAVIR	>15 mm	Yes
Liu[23], 2012, China	114 (80%)	38.32±11.36	same time	METAVIR	15∼20 mm	unclear
Wang[26], 2013, China	149 (93%)	37 (30∼42)	≤2d	Scheuer	>10 mm	Yes
Xun[28], 2013, China	197 (76%)	31 (21–45)	same time	Scheuer	>15 mm	unclear
Zhang[32], 2009, China	86 (60%)	39 (16–64)	<1d	METAVIR	15∼20 mm	unclear
Zhang[22], 2012, China	361 (62%)	36±11	≤7d	Scheuer	unclear	unclear
Zhang[31], 2010, China	212 (88%)	31±7	1day	Scheuer	20 mm	Yes

**Figure 2:**
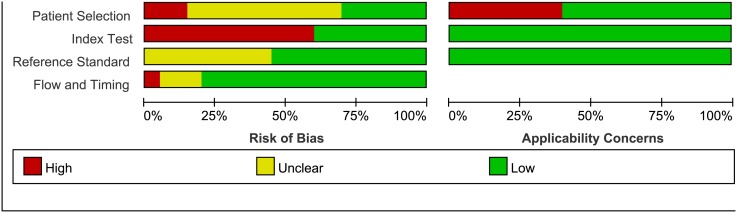
Methodological quality graph. Summary of methodological quality of studies according to Quality Assessment of Diagnostic Accuracy Studies-2 (QUADAS-2) tool concerning risk of bias and applicability in review authors’ judgments about each domain for each included study and review authors’ judgments about each domain, presented as percentages across included studies.

A cumulative bar plot of risk of bias and applicability concerns across all studies derived from QUADAS-2 was constructed (Figure 2). Unfortunately, a few studies stated that a consecutive or random sample of patients were enrolled, so there were not enough studies to do further subgroup analysis or sensitivity analysis. Despite this limitation, these factors were assessed in meta-regression for exploring sources of heterogeneity. The disease spectrum of 9 studies [11], [17], [19]–[21], [24], [26]–[28] were not in good accordance with our study and were excluded for further sensitivity analysis. Specifically, three of these studies [20], [24], [27] focused on patients with limited ALT (normal or less than 2× upper limit of normal), one focused on Hepatitis B virus e antigen (HBeAg)-positive patients [28], one focused on HBeAg-negative patients [26], one defined the urea nitrogen limitation when collecting samples [11], one included patients after therapy [19], one only included inpatients [17], and one did not describe the objective of the study clearly [21]. The bias of index test was mainly because many studies didn’t predefine the cutoff value. Five studies were found to have a disease progression bias [18], [19], [21], [29], [30], and nine studies did not describe whether interpretation of liver biopsy specimens was blinded to other test results [16], [17], [21]–[23], [28]–[30], [32].

### Diagnostic accuracy of the FIB-4 index for predicting significant fibrosis

**Figure 3:**
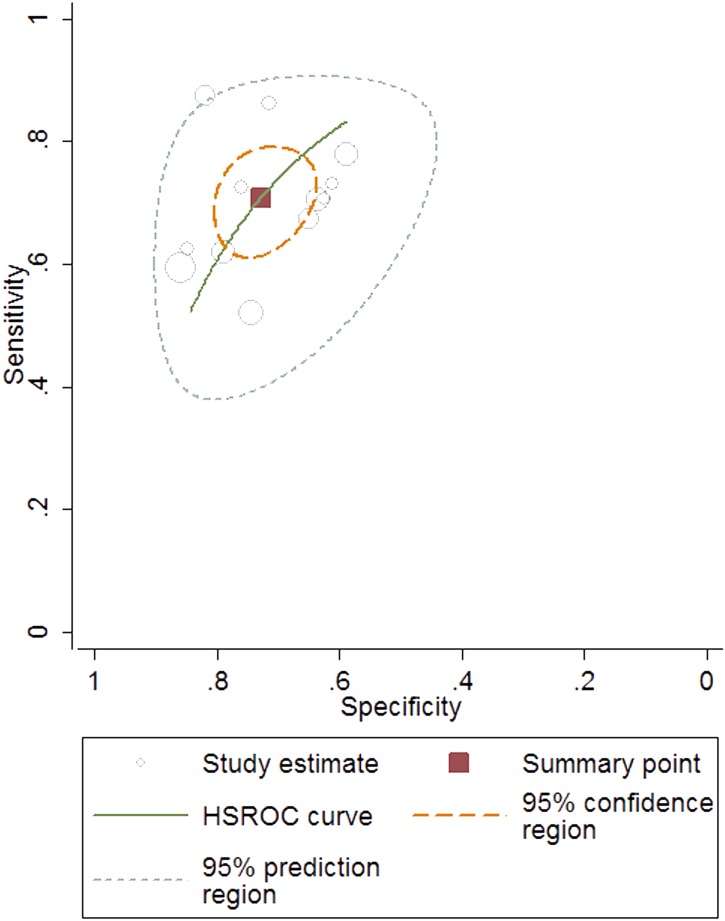
The hierarchical summary receiver operating characteristic (HSROC) curve of FIB-4 index for predicting significant fibrosis. The size of circles indicates the weight of the individual studies. The marked point on the curve represents the summary sensitivity and specificity. The area delimited by dashed line represents 95% confidence interval of the summary estimate. The area delimited by the dots represents the 95% prediction region, within which there is a 95%confidence that the true sensitivity and specificity of a future study should lie.

**Figure 4:**
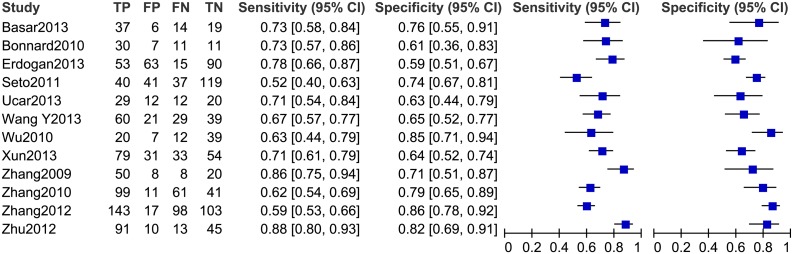
The forest plot of the FIB-4 index for predicting significant fibrosis.

Twelve studies, including 1,908 patients (male: 71%; average age: 37.1 years; average prevalence 57.4%) were used in our meta-analysis for testing the diagnostic accuracy of the FIB-4 index for predicting significant fibrosis (i.e. METAVIR F2–F4 vs. F0–F1; Table 2). The area under the HSROC was 0.78 (95% CI = 0.74–0.81; Figure 3). The summary sensitivity and specificity were 0.71 (95% CI = 0.64–0.77) and 0.73 (95% CI = 0.67–0.78), respectively (Figure 4). The heterogeneity was significant (*I^2^* = 94%), and the meta-regression showed that disease spectrum (*P* = 0.00) and blindness (*P* = 0.05) lead to the heterogeneity. The diagnostic performance of the FIB-4 index was improved after excluding studies with improper disease spectrum (Table 2). Although the data derived from blinded tests was more reliable, it was understandable that the diagnostic performance of the non-blinded subgroup (AUHSROC = 0.83) was better than that of the blinded subgroup (AUHSROC = 0.73). We also performed a subgroup analysis based on different cutoff values (Table 3). Based on the highest AUHSROC, the most appropriate cutoff value for detecting significant fibrosis was between 1.45 and 1.62, with a PLR of 2.83, and a NLR of 0.45. This means that patients with significant fibrosis have about 3-fold higher chance of being FIB-4 positive (above 1.62) compared with patients without significant fibrosis. If the FIB-4 was below the cutoff value, the probability that the patient has significant fibrosis was 45%. Thus, the FIB-4 index with a cutoff value between 1.45 and 1.62 was not suitable as a test for excluding the presence of significant fibrosis. Additionally, when a cutoff value of 3.25 is used, the PLR (6.04, 95% CI = 2.61–13.96) of the FIB-4 index is high enough to be used as a test to identify significant fibrosis. As none of subgroups (Table 3) has a sufficiently low NLR to be used to exclude significant fibrosis, FIB-4 index has suboptimal accuracy in excluding significant fibrosis.

**Table 2:** The diagnostic performance of the FIB-4 index for detecting significant fibrosis, severe fibrosis and cirrhosis.

Study characteristics		Number ofstudies		Summary estimates (95% CI)						Likelihood ratio (95% CI)				AUHSROC(95% CI)
				Sensitivity		Specificity		DOR		PLR		NLR		
***Significant fibrosis***														
All	12		0.71 (0.64–0.77)		0.73 (0.67–0.78)		6.52 (4.53–9.39)		2.61 (2.12–3.21)		0.4 (0.32 to 0.49)		0.78 (0.74–0.81)	
Exclude improper spectrumΔ	7		0.73 (0.63–0.81)		0.79 (0.73–0.84)		10.31 (6.52–16.30)		3.52 (2.75–4.51)		0.341 (0.25–0.47)		0.83 (0.80–0.86)	
Blindness*	7		0.68 (0.61–0.74)		0.68 (0.61–0.73)		4.39 (3.3–5.82)		2.09 (1.78–2.45)		0.48 (0.40–0.57)		0.73 (0.69–0.77)	
Non blindness	5		0.75 (0.62–0.84)		0.78 (0.69–0.85)		10.74 (5.60–20.59)		3.45 (2.39–4.98)		0.32 (0.21–0.49)		0.83 (0.80–0.86)	
Proper interval 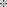	8		0.69 (0.62–0.75)		0.71 (0.64–0.78)		5.39 (4.02–7.22)		2.38 (1.95–2.90)		0.44 (0.37–0.52)		0.76 (0.72–0.79)	
Improper interval	4		0.76 (0.64–0.84)		0.75 (0.63–0.84)		9.45 (4.02–22.2)		3.04 (1.92–4.83)		0.32 (0.20–0.51)		0.82 (0.78–0.85)	
Exclude repeat patient#	11		0.69 (0.63–0.75)		0.73 (0.66–0.79)		6.09 (4.23–8.76)		2.56 (2.06–3.19)		0.42 (0.35–0.51)		0.77 (0.73–0.81)	
***Severe fibrosis***														
All	6		0.76 (0.64–0.85)		0.74 (0.70–0.79)		9.14 (5.35–15.60)		2.96 (2.48–3.53)		0.32 (0.21–0.49)		0.79 (0.75–82)	
Exclude improper spectrumΔ	5		0.79 (0.67–0.87)		0.72 (0.68–0.75)		9.17 (4.91–17.1)		2.76 (2.28–3.33)		0.3 (0.19–0.48)		0.73 (0.69–0.77)	
Blindness*	5		0.76 (0.62–0.86)		0.75 (0.70–0.79)		9.54 (5.18–17.58)		3.03 (2.50–3.68)		0.32 (0.19–0.52)		0.79 (0.76–0.83)	
***Cirrhosis***														
All	10		0.83 (0.78–0.88)		0.80 (0.73–0.86)		20.64 (11.54–36.93)		4.26 (3.04–5.96)		0.21 (0.15–0.28)		0.89 (0.85–0.91)	
Exclude improper spectrumΔ	6		0.85 (0.80–0.89)		0.77 (0.7–0.83)		19.10 (10.41–35.03)		3.75 (2.72–5.16)		0.2 (0.14–0.27)		0.88 (0.85–0.91)	
Blindness*	5		0.80 (0.7–0.87)		0.77 (0.72–0.82)		13.25 (6.5–27.05)		3.48 (2.59–4.69)		0.26 (0.17–0.41)		0.85 (0.81–0.88)	
Non blindness	5		0.85 (0.80–0.90)		0.85 (0.72–0.92)		32.53 (14.48–73.08)		5.6 (3–10.57)		0.17 (0.12–0.25)		0.87 (0.84–0.90)	
Proper interval 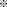	8		0.83 (0.77–0.88)		0.80 (0.72–0.86)		20.04 (10.32–38.92)		4.18 (2.9–6.04)		0.21 (0.15–0.30)		0.88 (0.85–0.91)	
Exclude repeat patient#	9		0.83 (0.77–0.88)		0.82 (0.75–0.87)		21.93 (11.75–40.95)		4.52 (3.15–6.47)		0.21 (0.15–0.29)		0.89 (0.86–0.91)	

Δ The disease spectrum of some studies [11], [17], [19]–[21], [24], [26]–[28] were not in good accordance with our study, thus they were excluded for sensitivity analysis.

*The studies in which the reference standard results were interpreted without knowledge of the results of the index tests, were grouped into “Blindness”.

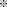
The “proper interval” was defined as the time interval between the determination of the FIB-4 index and liver biopsy was no longer than 7 days.

# As two studies [22], [32] were written by the same author, the study [32] with the smaller sample size was excluded for sensitivity analysis. 95% CI: 95% confidence interval. DOR: diagnostic odds ratio. PLR: positive likelihood ratio; NLR: negative likelihood ratio. AUHSROC: area under the hierarchical summary receiver operating characteristic curve.

**Table 3:** Subgroup analysis based on different FIB-4 index cutoff values.

Thresholds	Number ofstudies	Summary estimates(95% CI)			Likelihood ratio (95%CI)		AUHSROC(95% CI)
		Sensitivity	Specificity	DOR	PLR	NLR	
***Significant fibrosis***							
0.8–1.085	5	0.73 (0.68–0.77)	0.62 (0.56–0.67)	4 (3–6)	1.9 (1.6–2.2)	0.44 (0.36–0.54)	0.73 (0.69–0.77)
1.45–1.62	6	0.65 (0.56–0.73)	0.77 (0.7–0.83)	6.24 (4.06–9.61)	2.83 (2.16–3.71)	0.45 (0.36–0.57)	0.78 (0.74–0.81)
3.25	3	0.18 (0.13–0.24)	0.98 (0.95–0.99)	7.53 (3.06–18.54)	6.04 (2.61–13.96)	0.85 (0.76–0.95)	0.61 (0.57–0.65)
***Severe fibrosis***							
1.45–1.65	4	0.68 (0.6–0.75)	0.75 (0.69–0.81)	6.44 (4.44–9.35)	2.75 (2.21–3.43)	0.42 (0.34–0.53)	0.77 (0.73–0.80)
***Cirrhosis***							
1.6–2.29	7	0.82 (0.75–0.88)	0.77 (0.71–0.82)	15.55 (8.69–27.80)	3.59 (2.73–4.74)	0.23 (0.16–0.33)	0.87 (0.84–0.90)
2.9–3.6	3	0.42 (0.36–0.48)	0.96 (0.95–0.97)	46.28 (18.3–117.04)	13.38 (9.24–19.37)	0.3 (0.06–0.45)	0.96 (0.92–1.00)

95% CI: 95% confidence interval. DOR: diagnostic odds ratio. PLR: positive likelihood ratio; NLR: negative likelihood ratio. AUHSROC: area under the hierarchical summary receiver operating characteristic curve.

### Diagnostic accuracy of the FIB-4 index for predicting severe fibrosis

**Figure 5:**
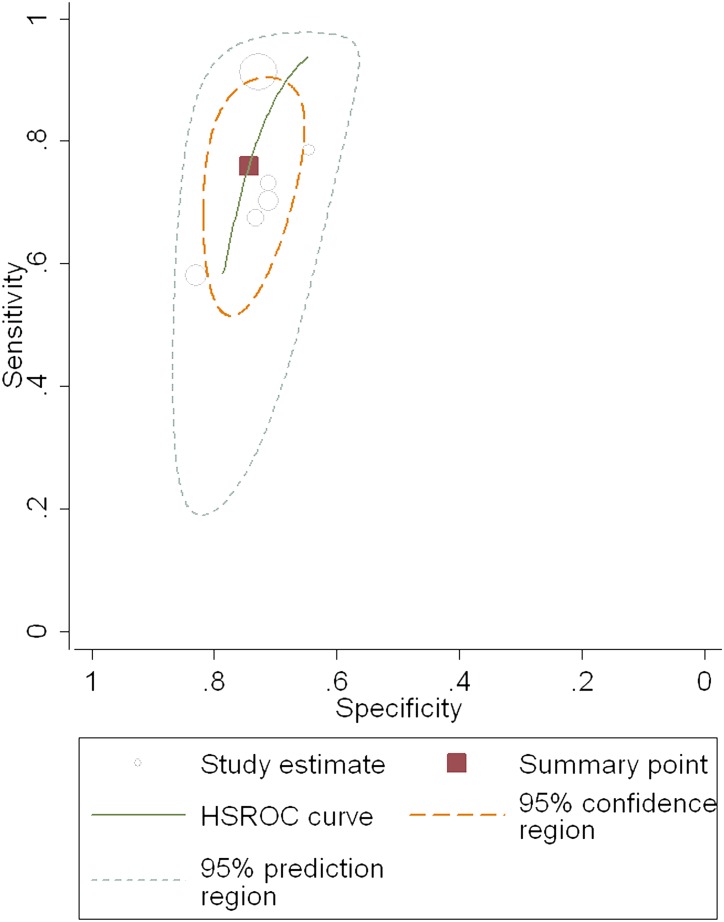
The hierarchical summary receiver operating characteristic (HSROC) curve of FIB-4 index for predicting severe fibrosis. The size of circles indicates the weight of the individual studies. The marked point on the curve represents the summary sensitivity and specificity. The area delimited by dashed line represents 95% confidence interval of the summary estimate. The area delimited by the dots represents the 95% prediction region, within which there is a 95% confidence that the true sensitivity and specificity of a future study should lie.

**Figure 6:**
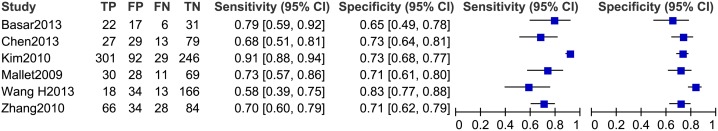
The forest plot of the FIB-4 index for predicting severe fibrosis.

Six studies of 1,473 patients (male: 68.9%; average age: 37.3 years, average prevalence: 33.5%) were involved in the meta-analysis for testing the diagnostic accuracy of the FIB-4 index for predicting severe fibrosis (i.e., METAVIR F3–F4 vs. F0–F2). The cutoff values ranged from 1 to 3.25. The area under the HSROC was 0.79 (95% CI = 0.75-82; Figure 5). The summary sensitivity and specificity were 0.76 (95% CI = 0.64–0.85) and 0.74 (95% CI = 0.70–0.79), respectively (Figure 6). The heterogeneity was significant (*I^2^* = 93%), and the meta-regression showed that disease spectrum (*P* = 0.01) and prevalence (*P* = 0.01) lead to the heterogeneity. There was only one study with improper disease spectrum [26]. At the same time, disease prevalence of this study (prevalence = 0.13) was also much lower than that of the others. After excluding this study, the area under HSROC dropped from 0.79 to 0.73. Subgroup analysis (Table 3) showed that in four studies with cutoff values ranging from 1.45 to 1.65, the area under the summary receiver-operating characteristic curve (AUROC) was 0.77 (95% CI = 0.73–0.80). The corresponding PLR (2.75, 95% CI = 2.21–3.43) and NLR (0.42, 95% CI = 0.34–0.53) indicated that using the FIB-4 index with a cutoff value between 1.45 and 1.65 has a suboptimal accuracy in identifying and excluding severe fibrosis.

### Diagnostic accuracy of the FIB-4 index for predicting cirrhosis

**Figure 7:**
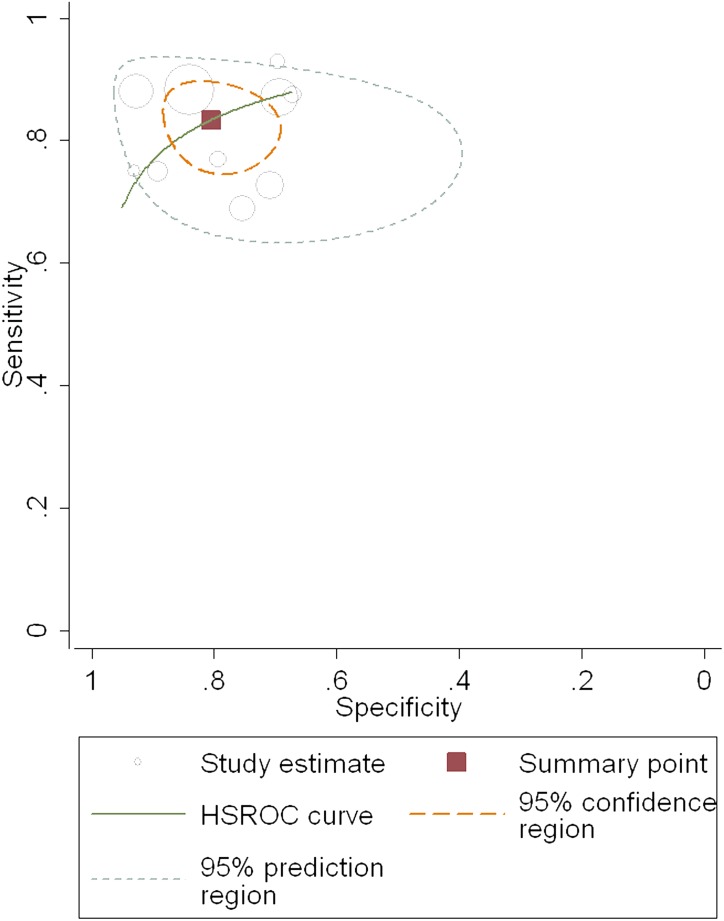
The hierarchical summary receiver operating characteristic (HSROC) curve of FIB-4 index for predicting cirrhosis. The size of circles indicates the weight of the individual studies. The marked point on the curve represents the summary sensitivity and specificity. The area delimited by dashed line represents 95% confidence interval of the summary estimate. The area delimited by the dots represents the 95% prediction region, within which there is a 95% confidence that the true sensitivity and specificity of a future study should lie.

**Figure 8:**
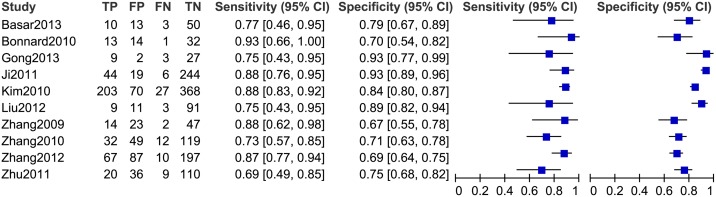
The forest plot of the FIB-4 index for predicting cirrhosis.

Ten studies of 2,105 patients (male: 69.9%, average age: 37.9 years, average prevalence of cirrhosis 20.8%) were involved in the meta-analysis for testing the diagnostic accuracy of the FIB-4 index for predicting cirrhosis (i.e., METAVIR F4 vs. F0–F3). The cutoff values ranged from 1.05 to 3.6, and the area under the HSROC was 0.89 (95% CI = 0.85–0.91; Figure 7), so the diagnostic performance was nearly excellent [42], [43]. The summary sensitivity and specificity were 0.83 (95% CI = 0.78–0.88) and 0.80 (95% CI = 0.73–0.86), respectively (Figure 8). The heterogeneity was significant (*I^2^* = 67%), and the meta-regression showed that the potential factors described above didn’t lead to the heterogeneity (*P*>0.05). The most appropriate cutoff value for detecting significant fibrosis was between 2.9 and 3.6. At this range, the AUROC was 0.96 (95% CI = 0.92–1.00), so it was classified as excellent. The PLR (13.38, 95% CI = 9.24–19.37) for the FIB-4 index was high enough to be used to identify cirrhosis, although the NLR (0.3, 95% CI = 0.06–0.45) was not low enough to exclude cirrhosis.

### Publication bias

A publication bias was not detected when tested using Egger’s test or the trim and fill method (Table 4). For Egger’s test, the publication bias 95% CI of every group included zero and the *P*-value was >0.05, so there was no statistical difference between publication bias and zero. This means that no publication bias was present. For the trim and fill method, the summary diagnostic odds ratio (SDOR) was always >1 both before and after trim and fill, meaning that trimming and filling studies didn’t influence the outcome of the meta-analysis (SDOR always >1). Taken together this means that publication bias was nonexistent in our meta-analysis.

**Table 4:** Analysis of publication bias for studies.

Staging	P value of bias	95%CI of bias	SDOR after trim and fill
Significant fibrosis	0.18	(−1.34∼6.26)	6.394 (4.44∼9.21)
Severe fibrosis	0.08	(−12.56∼1.07)	8.38 (4.27∼16.43)
Cirrhosis	0.75	(−3.93∼2.94)	19.67 (10.53∼36.74)

95% CI: 95% confidence interval. SDOR: summary diagnostic odds ratio.

## Discussion

Accurate diagnosis of liver fibrosis is clinically advantageous. Liver biopsy is the gold standard for diagnosing fibrosis; however, its clinical application is hampered by various limitations. Despite these limitations, an ideal alternative to liver biopsies has not been found [67], [68]. In this meta-analysis we assessed the diagnostic accuracy of the FIB-4 index as a non-invasive alternative to liver biopsy.

The FIB-4 index is a simple and inexpensive noninvasive marker of liver fibrosis. Recently, the diagnostic value of the FIB-4 index in predicting the extent of fibrosis has been substantiated, and is even considered by some to be the best noninvasive index [11], [13]; however, others have highlighted its weaknesses [10], [26]. The current study comprehensively analyzed the predictive power of the FIB-4 index using a meta-analysis of previously published studies. The area under the HSROC for the FIB-4 index was 0.78, and 0.79 and 0.89 for predicting significant and severe fibrosis, and cirrhosis, respectively. Thus, the summary diagnostic performance of FIB-4 for significant and severe fibrosis was nearly good, and for cirrhosis was nearly excellent.

As the summary estimates of all cutoff values was deemed difficult to interpret and use in clinical practice, a subgroup analysis based on different cutoff values was performed. The recommended cutoff value for predicting significant fibrosis was between 1.45 and 1.62 based on the highest AUHSROC, but it still had suboptimal accuracy in excluding significant fibrosis. Fortunately, we found that the FIB-4 index with a cutoff value of 3.25 was suitable for identifying significant fibrosis. For severe fibrosis, the recommended cutoff value was between 1.45 and 1.65, and it has a suboptimal accuracy in identifying and excluding severe fibrosis. For cirrhosis, the recommended cutoff value was between 2.9 and 3.6, and the diagnostic performance was excellent (AUROC = 0.96). Thus, patient’s with a FIB-4 index above 3.6 can almost be diagnosed with cirrhosis, with a PLR = 13.38.

In terms of other noninvasive indexes, the APRI has the advantage of including only two inexpensive laboratory tests, which are performed routinely, and the FibroTest/Fibrosure is one of the most investigated and most frequently used tools for assessing liver fibrosis. The diagnostic performance of these two non-invasive indexes has been evaluated by meta-analysis [67], [69]. If we compare our meta-analysis of the FIB-4 index with these studies we can see that for significant fibrosis, AUROC of the APRI and FibroTest/Fibrosure was 0.79 (SE = 0.0243) and 0.84 (95% CI = 0.78–0.88), respectively. Thus, the diagnostic accuracy of the FIB-4 was similar to that of the APRI, and worse than the FibroTest/Fibrosure. For cirrhosis, the AUROC of the APRI and FibroTest/Fibrosure was 0.75 (SE = 0.0237) and 0.87 (95% CI = 0.85–0.90), respectively. Thus, based on our meta-analysis, the FIB-4 index was superior to that of the APRI, and similar to that of the FibroTest/Fibrosure for diagnosing cirrhosis. Additionally, another meta-analysis revealed that the AUROC of the FIB-4 index for significant fibrosis and cirrhosis with HCV infection was 0.74 and 0.87, respectively [70]. Thus, the diagnostic value of the FIB-4 index for predicting HBV-related fibrosis was also slightly better than that for HCV, although it was originally applied to HCV and HIV co-infection [14], [15]. Unfortunately, meta-analyses of other non-invasive tests for predicting HBV related fibrosis was not found, so comparison with the FIB-4 index was not possible.

There are two strengths to the current meta-analysis. First, although the diagnostic performance of the FIB-4 index for HBV-related fibrosis has previously been assessed by several studies [10], [11], [13], [16]–[32], our evaluation combined the data from previously published work in a meta-analysis, thereby strengthening its accuracy. Second, we searched the CNKI and CBMdisc databases that provided authoritative and comprehensive data from Chinese populations. This is important because the prevalence of HBV infection is much higher than that of HCV infection in Chinese populations [71]. Additionally, we found that of the 15 eligible studies written in English for this meta-analysis [10], [11], [13], [16], [18]–[20], [23]–[30], nine were written by Chinese groups [10], [20], [23], [24], [26]–[30]. Thus, authors not searching Chinese databases may have overlooked some valuable studies.

There are three limitations to the current meta-analysis. First, there were 20 eligible studies included in the meta-analysis, but this number was too small for further subgroup analysis. This limitation was compounded by the fact that there were few studies with a large sample size and multiple centers. The second limitation was the significant heterogeneity of included studies. A considerable variation between the results of diagnostic studies is a common occurrence, possibly to a greater extent than is seen for therapeutic interventions [72]. One of the potential sources of heterogeneity and a direct consequence of the fact that the importance of rigorous design has been less well appreciated for diagnostic studies than for therapeutic interventions, is poor adherence to methodological constraints [69], [73]. This is noticeable in many studies that we included, and can be considered as a general problem in many studies dealing with the diagnostic accuracy of liver fibrosis markers, as already noted by others [73]. In our study, although disease spectrum, blindness and prevalence were found to be the factors causing heterogeneity, and further sensitivity analysis and/or subgroup analysis were performed in our study, more detailed subgroup analysis, such as grouping by both proper disease spectrum and blindness, is needed. Unfortunately, the eligible studies were too few to perform this. Finally, we only included published manuscripts, so bias in the selection of search channels may have influenced our results.

Our meta-analysis has several implications for future research. For example, we believe that more studies on the diagnostic accuracy for liver fibrosis are needed in patient populations with CHB. In the future, authors of studies exploring the performance of the FIB-4 index in CHB patients should be encouraged to insist on a rigorous design and methodology. In this regard, QUADAS-2 [36] describes what is required for a rigorous study design and methodology, and is a good tool for guiding diagnostic study design. As common flaws in design and methodology found in our eligible studies, we emphasize two points: first, a study should ideally enroll all consecutive, or a random sample of, eligible patients with suspected disease – otherwise there is potential for bias. Second, selecting the test threshold to optimize sensitivity and/or specificity may lead to overoptimistic estimates of test performance, which is likely to be poorer in an independent sample of patients in whom the same threshold is used [74]. As a result, if a threshold was used, it should be pre-specified.

Implications for practice deriving from our results suggest that the FIB-4 index is of excellent utility for detecting cirrhosis in patients with CHB, and has moderate accuracy in detecting significant fibrosis. On the other hand, it has suboptimal performance in the exclusion of significant and severe fibrosis, and cirrhosis. Thus, it is necessary to further improve the test or combine it with other noninvasive modalities in order to improve its accuracy.

## Supporting Information

File S1 **Search strategy of Ovid database.** This literature search was performed in November 2013.
(DOC)

File S2 **Search strategy of Pubmed database.** This literature search was performed in November 2013.
(CSV)

Checklist S1 **PRISMA 2009 checklist.**
(DOC)
