# Supplementary material for: The Diagnostic Value of the FIB-4 Index for Staging Hepatitis B-Related Fibrosis: A Meta-Analysis
Source: PLoS One. 2014 Aug 28;9(8):e105728. doi: 10.1371/journal.pone.0105728 (PMC4148327; doi:10.1371/journal.pone.0105728)
Supplement: File S1 — Search strategy of Ovid database. This literature search was performed in November 2013. (DOC) [file pone.0105728.s001.doc]

Database: Books@Ovid <December 03, 2013>, Journals@Ovid, Zhejiang University Full-Text Journals(LWW Journals), PsycARTICLES Full Text Journals, BIOSIS Previews <1994 to 2011>, CAB Abstracts + Full Text, ERIC <1965 to July 2013>, Food Science and Technology Abstracts <1969 to 2013 December Week 1>, PsycINFO <1806 to November Week 4 2013>

Search Strategy:

--------------------------------------------------------------------------------

1 hepatitis B.mp. [mp=tx, bt, bo, ti, ab, ct, mc, st, or, tn, ps, ds, cb, rn, sq, mq, ge, tm, mi, sh, bc, cc, gl, gn, ot, hw, id, tc] (122673)

2 FIB-4.ab. (147)

3 1 and 2 (65)

4 (fibrosis or cirrhosis).ab. (119769)

5 3 and 4 (63)

***************************

1.

Serum hepatitis B surface antigen quantification as a useful assessment for significant fibrosis in hepatitis B e antigen-positive hepatitis B virus carriers.

Xun, Yun-hao 2,3; Zang, Guo-qing 2; Guo, Jian-chun 3; Yu, Xiu-li 3; Liu, Hong 3; Xiang, Jing 4; Liu, Jing 4; Shi, Jun-ping 3,*

Journals@Ovid

Journal of Gastroenterology & Hepatology. 28(11):1746-1755, November 2013.

[Miscellaneous Article] [Hepatology: Clinical Hepatology]

AN: 00001753-201311000-00013.

<td colspan="">

Link to the Ovid Full Text or citation:

[Click here for full text options](http://ovidsp.ovid.com/ovidweb.cgi?T=JS&CSC=Y&NEWS=N&PAGE=fulltext&D=ovfto&AN=00001753-201311000-00013)

2.

APRI, the FIB-4 score, and Forn's index have noninvasive diagnostic value for liver fibrosis in patients with chronic hepatitis B.

Ucar, Fatma a; Sezer, Sevilay d; Ginis, Zeynep a; Ozturk, Gulfer a; Albayrak, Aynur b; Basar, Omer c; Ekiz, Fuat c; Coban, Sahin c; Yuksel, Osman c; Armutcu, Ferah e; Akbal, Erdem f

Journals@Ovid

European Journal of Gastroenterology & Hepatology. 25(9):1076-1081, September 2013.

[Article] [Original Articles: Hepatitis]

AN: 00042737-201309000-00010.

<td colspan="">

Link to the Ovid Full Text or citation:

[Click here for full text options](http://ovidsp.ovid.com/ovidweb.cgi?T=JS&CSC=Y&NEWS=N&PAGE=fulltext&D=ovfto&AN=00042737-201309000-00010)

3.

Impact of Lifetime Alcohol Use on Liver Fibrosis in a Population of HIV-Infected Patients With and Without Hepatitis C Coinfection.

Fuster, Daniel 1; Tsui, Judith I. 1; Cheng, Debbie M. 1,2; Quinn, Emily K. 3; Bridden, Carly 1; Nunes, David 4; Libman, Howard 5; Saitz, Richard 1,6; Samet, Jeffrey H. 1,7

Journals@Ovid

Alcoholism: Clinical & Experimental Research. 37(9):1527-1535, September 2013.

[Article] [Original Article: Epidemiology, Diagnosis & Comorbidity]

AN: 00078240-201309000-00011.

<td colspan="">

Link to the Ovid Full Text or citation:

[Click here for full text options](http://ovidsp.ovid.com/ovidweb.cgi?T=JS&CSC=Y&NEWS=N&PAGE=fulltext&D=ovfto&AN=00078240-201309000-00011)

4.

The role of transient elastography in the detection of liver disease in patients with chronic pancreatitis.

Frossard, Jean Louis 1; Giostra, Emiliano 1; Rubbia-Brandt, Laura 2; Hadengue, Antoine 1; Spahr, Laurent 1

Journals@Ovid

Liver International. 33(7):1121-1127, August 2013.

[Miscellaneous Article] [Biomarkers]

AN: 00137700-201308000-00018.

<td colspan="">

Link to the Ovid Full Text or citation:

[Click here for full text options](http://ovidsp.ovid.com/ovidweb.cgi?T=JS&CSC=Y&NEWS=N&PAGE=fulltext&D=ovfto&AN=00137700-201308000-00018)

5.

Noninvasive Serum Fibrosis Markers for Screening and Staging Chronic Hepatitis C Virus Patients in a Large US Cohort.

Holmberg, Scott D. 1; Lu, Mei 2; Rupp, Loralee B. 3; Lamerato, Lois E. 3; Moorman, Anne C. 1; Vijayadeva, Vinutha 4; Boscarino, Joseph A. 5; Henkle, Emily M. 6; Gordon, Stuart C. 2; for the Chronic Hepatitis Cohort Study (CHeCS) Investigatorsa; Holmberg, Scott D.; Teshale, Eyasu H.; Spradling, Philip R.; Moorman, Anne C.; Gordon, Stuart C.; Nerenz, David R.; Lu, Mei; Lamerato, Lois; Rupp, Loralee B.; Akkerman, Nonna; Oja-Tebbe, Nancy J.; Cogan, Chad M.; Larkin, Dana; Boscarino, Joseph A.; Daar, Zahra S.; Smith, Robert E.; Curry, Patrick J.; Geise, Brandon D.; Leader, Joe B.; Nakasato, Cynthia C.; Vijayadeva, Vinutha; Sylva, Kelly E.; Parker, John V.; Schmidt, Mark M.; Henkle, Emily M.; Dodge, Tracy L.; Keast, Erin M.; Drew, Lois

Journals@Ovid

Clinical Infectious Diseases. 57(2):240-246, July 15, 2013.

[Article] [ARTICLES AND COMMENTARIES]

AN: 01451458-201307150-00014.

<td colspan="">

Link to the Ovid Full Text or citation:

[Click here for full text options](http://ovidsp.ovid.com/ovidweb.cgi?T=JS&CSC=Y&NEWS=N&PAGE=fulltext&D=ovfto&AN=01451458-201307150-00014)

6.

Evaluation of the Liver Fibrosis Index calculated by using real-time tissue elastography for the non-invasive assessment of liver fibrosis in chronic liver diseases.

Tomeno, Wataru 1; Yoneda, Masato 1; Imajo, Kento 1; Suzuki, Kaori 1; Ogawa, Yuji 1; Shinohara, Yoshiyasu 1; Mawatari, Hironori 1; Fujita, Koji 1; Shibata, Wataru 1; Kirikoshi, Hiroyuki 1; Maeda, Shin 1; Nakajima, Atsushi 1; Saito, Satoru 1

Journals@Ovid

Hepatology Research. 43(7):735-742, July 2013.

[Article] [Original Articles; Clinical hepatology]

AN: 00066974-201307000-00006.

<td colspan="">

Link to the Ovid Full Text or citation:

[Click here for full text options](http://ovidsp.ovid.com/ovidweb.cgi?T=JS&CSC=Y&NEWS=N&PAGE=fulltext&D=ovfto&AN=00066974-201307000-00006)

7.

Non-invasive tests for fibrosis and liver stiffness predict 5-year survival of patients chronically infected with hepatitis B virus.

de Ledinghen, V. 1,2; Vergniol, J. 1; Barthe, C. 1; Foucher, J. 1,3; Chermak, F. 1; Le Bail, B. 2,4; Merrouche, W. 1; Bernard, P.-H. 3

Journals@Ovid

Alimentary Pharmacology & Therapeutics. 37(10):979-988, May 2013.

[Article] [Original Article]

AN: 00001716-201305010-00007.

<td colspan="">

Link to the Ovid Full Text or citation:

[Click here for full text options](http://ovidsp.ovid.com/ovidweb.cgi?T=JS&CSC=Y&NEWS=N&PAGE=fulltext&D=ovftn&AN=00001716-201305010-00007)

8.

Prediction of significant fibrosis and cirrhosis in hepatitis B e-antigen negative patients with chronic hepatitis B using routine parameters.

Wang, Yan 1; Xu, Ming-Yi 1,*; Zheng, Rui-Dan 2; Xian, Jian-Chun 3; Xu, Hong-Tao 3; Shi, Jun-Ping 4; Li, Shi-Bo 5; Qu, Ying 1; Dong, Yu-Wei 1; Lu, Lun-Gen 1

Journals@Ovid

Hepatology Research. 43(5):441-451, May 2013.

[Article] [Original Article: Clinical hepatology]

AN: 00066974-201305000-00001.

<td colspan="">

Link to the Ovid Full Text or citation:

[Click here for full text options](http://ovidsp.ovid.com/ovidweb.cgi?T=JS&CSC=Y&NEWS=N&PAGE=fulltext&D=ovftn&AN=00066974-201305000-00001)

9.

Evaluation of seven noninvasive models in staging liver fibrosis in patients with chronic hepatitis B virus infection.

Ma, Jing; Jiang, Yongfang; Gong, Guozhong

Journals@Ovid

European Journal of Gastroenterology & Hepatology. 25(4):428-434, April 2013.

[Article] [Original Articles: Hepatitis]

AN: 00042737-201304000-00007.

<td colspan="">

Link to the Ovid Full Text or citation:

[Click here for full text options](http://ovidsp.ovid.com/ovidweb.cgi?T=JS&CSC=Y&NEWS=N&PAGE=fulltext&D=ovftn&AN=00042737-201304000-00007)

10.

Comparison of FIB-4 and APRI in Chinese HBV-infected patients with persistently normal ALT and mildly elevated ALT.

Wang, H. 1; Xue, L. 2; Yan, R. 1; Zhou, Y. 1; Wang, M. S. 1; Cheng, M. J. 1; Huang, H. J. 1

Journals@Ovid

Journal of Viral Hepatitis. 20(4):e3-e10, April 2013.

[Article] [ORIGINAL ARTICLES]

AN: 00043778-201304000-00012.

<td colspan="">

Link to the Ovid Full Text or citation:

[Click here for full text options](http://ovidsp.ovid.com/ovidweb.cgi?T=JS&CSC=Y&NEWS=N&PAGE=fulltext&D=ovftn&AN=00043778-201304000-00012)

11.

Association between noninvasive fibrosis markers and mortality among adults with nonalcoholic fatty liver disease in the United States.

Kim, Donghee 1,2; Kim, Ray W. 1,*; Kim, Hwa Jung 3; Therneau, Terry M. 4

Journals@Ovid

Hepatology. 57(4):1357-1365, April 2013.

[Miscellaneous Article] [Steatohepatitis/Metabolic Liver Disease]

AN: 01515467-201304000-00010.

<td colspan="">

Link to the Ovid Full Text or citation:

[Click here for full text options](http://ovidsp.ovid.com/ovidweb.cgi?T=JS&CSC=Y&NEWS=N&PAGE=fulltext&D=ovftn&AN=01515467-201304000-00010)

12.

Diagnostic potential of serum direct markers and non-invasive fibrosis models in patients with chronic hepatitis B.

Gumusay, Ozge 1; Ozenirler, Seren 2; Atak, Aysegul 3; Sonmez, Cemile 3; Ozkan, Secil 4; Tuncel, Ayse Ftnat 5[iota]; Ylmaz, Guldal 6[iota]; Akyol, Gulen 6

Journals@Ovid

Hepatology Research. 43(3):228-237, March 2013.

[Article] [Original Article: Clinical hepatology]

AN: 00066974-201303000-00002.

<td colspan="">

Link to the Ovid Full Text or citation:

[Click here for full text options](http://ovidsp.ovid.com/ovidweb.cgi?T=JS&CSC=Y&NEWS=N&PAGE=fulltext&D=ovftn&AN=00066974-201303000-00002)

13.

Noninvasive estimation of fibrosis progression overtime using the FIB-4 index in chronic hepatitis C.

Tamaki, N. 1; Kurosaki, M. 1; Tanaka, K. 1; Suzuki, Y. 1; Hoshioka, Y. 1; Kato, T. 1; Yasui, Y. 1; Hosokawa, T. 1; Ueda, K. 1; Tsuchiya, K. 1; Nakanishi, H. 1; Itakura, J. 1; Asahina, Y. 1; Izumi, N. 1

Journals@Ovid

Journal of Viral Hepatitis. 20(1):72-76, January 2013.

[Article] [ORIGINAL ARTICLES]

AN: 00043778-201301000-00009.

<td colspan="">

Link to the Ovid Full Text or citation:

[Click here for full text options](http://ovidsp.ovid.com/ovidweb.cgi?T=JS&CSC=Y&NEWS=N&PAGE=fulltext&D=ovftn&AN=00043778-201301000-00009)

14.

Suboptimal performance of simple noninvasive tests for advanced fibrosis in Chinese patients with nonalcoholic fatty liver disease.

Xun, Yun Hao 1,2; Fan, Jian Gao 3; Zang, Guo Qing 1; Liu, Hong 2; Jiang, Yan Ming 2; Xiang, Jing 2; Huang, Qian 2; Shi, Jun Ping 2

Journals@Ovid

Journal of Digestive Diseases. 13(11):588-595, November 2012.

[Article] [Original article]

AN: 01337992-201211000-00006.

<td colspan="">

Link to the Ovid Full Text or citation:

[Click here for full text options](http://ovidsp.ovid.com/ovidweb.cgi?T=JS&CSC=Y&NEWS=N&PAGE=fulltext&D=ovftn&AN=01337992-201211000-00006)

15.

Serum levels of tissue inhibitor of metalloproteinase-1 are correlated with liver fibrosis in patients with chronic hepatitis B.

Zhu, Chuan Long 1; Li, Wen Ting 1; Li, Yi 1; Gao, Ren Tao 1

Journals@Ovid

Journal of Digestive Diseases. 13(11):558-563, November 2012.

[Article] [Original article]

AN: 01337992-201211000-00002.

<td colspan="">

Link to the Ovid Full Text or citation:

[Click here for full text options](http://ovidsp.ovid.com/ovidweb.cgi?T=JS&CSC=Y&NEWS=N&PAGE=fulltext&D=ovftn&AN=01337992-201211000-00002)

16.

LecT-hepa, a glyco-marker derived from multiple lectins, as a predictor of liver fibrosis in chronic hepatitis C patients.

Ito, Kiyoaki 1; Kuno, Atsushi 2; Ikehara, Yuzuru 2; Sugiyama, Masaya 1; Saito, Hiroaki 1; Aoki, Yoshihiko 1; Matsui, Teppei 1; Imamura, Masatoshi 1; Korenaga, Masaaki 1; Murata, Kazumoto 1; Masaki, Naohiko 1; Tanaka, Yasuhito 3; Hige, Shuhei 4; Izumi, Namiki 5; Kurosaki, Masayuki 5; Nishiguchi, Shuhei 6; Sakamoto, Michiie 7; Kage, Masayoshi 8; Narimatsu, Hisashi 2; Mizokami, Masashi 1*#

Journals@Ovid

Hepatology. 56(4):1448-1456, October 2012.

[Miscellaneous Article] [Liver Failure/Cirrhosis/Portal Hypertension]

AN: 01515467-201210000-00029.

<td colspan="">

Link to the Ovid Full Text or citation:

[Click here for full text options](http://ovidsp.ovid.com/ovidweb.cgi?T=JS&CSC=Y&NEWS=N&PAGE=fulltext&D=ovftn&AN=01515467-201210000-00029)

17.

The Accuracy of Noninvasive Methods in Predicting the Development of Hepatocellular Carcinoma and Hepatic Decompensation in Patients With Chronic Hepatitis B.

Chon, Young Eun MD *,+,++; Jung, Eun Suk MD *,+,++; Park, Jun Yong MD *,+,++,[S]; Kim, Do Young MD, PhD *,+,++,[S]; Ahn, Sang Hoon MD, PhD *,+,++,[S]; Han, Kwang-Hyub MD, PhD *,+,++,[S]; Chon, Chae Yoon MD, PhD *,+,++,[S]; Jung, Kyu Sik MD *,+,++; Kim, Seung Up MD *,+,++,[S]

Journals@Ovid

Journal of Clinical Gastroenterology. 46(6):518-525, July 2012.

[Article] [LIVER, PANCREAS AND BILIARY TRACT: Original Article]

AN: 00004836-201207000-00016.

<td colspan="">

Link to the Ovid Full Text or citation:

[Click here for full text options](http://ovidsp.ovid.com/ovidweb.cgi?T=JS&CSC=Y&NEWS=N&PAGE=fulltext&D=ovftn&AN=00004836-201207000-00016)

18.

Assessing mortality in women with hepatitis C virus and HIV using indirect markers of fibrosis.

Bambha, Kiran a; Pierce, Christopher b; Cox, Christopher b; French, Audrey L. c; Tien, Phyllis C. a,i; Sharp, Gerald B. d; Augenbraun, Michael e; Glesby, Marshall J. f; Villacres, Maria C. g; Plankey, Michael h; Strickler, Howard D. j; Gange, Stephen J. b; Peters, Marion G. a

Journals@Ovid

AIDS. 26(5):599-607, March 13, 2012.

[Article] [CLINICAL SCIENCE]

AN: 00002030-201203130-00009.

<td colspan="">

Link to the Ovid Full Text or citation:

[Click here for full text options](http://ovidsp.ovid.com/ovidweb.cgi?T=JS&CSC=Y&NEWS=N&PAGE=fulltext&D=ovftm&AN=00002030-201203130-00009)

19.

Change in Fibrosis Score as a Predictor of Mortality Among HIV-Infected Patients with Viral Hepatitis.

Jain, Mamta K. M.D., M.P.H. 1; Seremba, Emmanuel MBBS 1; Bhore, Rafia Ph.D. 2; Dao, Doan B.S. 1; Joshi, Reeti MBBS 1; Attar, Nahid 1; Yuan, He-Jun MBBS, Ph.D. 1; Lee, William M. M.D. 1

Journals@Ovid

AIDS Patient Care & Stds. 26(2):73-80, February 2012.

[Miscellaneous Article] [CLINICAL AND EDIDEMIOLOGIC RESEARCH]

AN: 00063043-201202000-00003.

<td colspan="">

Link to the Ovid Full Text or citation:

[Click here for full text options](http://ovidsp.ovid.com/ovidweb.cgi?T=JS&CSC=Y&NEWS=N&PAGE=fulltext&D=ovftm&AN=00063043-201202000-00003)

20.

Comparison between different noninvasive fibrosis seromarkers and liver biopsy in staging fibrosis in Egyptian patients with chronic hepatitis C virus Infection.

Shaker, Mohamed Kamal; Khalifa, Mohamed Omar

Journals@Ovid

Egyptian Liver Journal. 2(1):12-15, January 2012.

[Article] [Original articles]

AN: 01438841-201201000-00003.

<td colspan="">

Link to the Ovid Full Text or citation:

[Click here for full text options](http://ovidsp.ovid.com/ovidweb.cgi?T=JS&CSC=Y&NEWS=N&PAGE=fulltext&D=ovftm&AN=01438841-201201000-00003)

21.

High plasma CXCL10 levels are associated with HCV-genotype 1, and higher insulin resistance, fibrosis, and HIV viral load in HIV/HCV coinfected patients.

Berenguer, Juan a; Fernandez-Rodriguez, Amanda b; Jimenez-Sousa, Maria Angeles b; Cosin, Jaime a; Zarate, Paola b,c; Micheloud, Dariela b,d; Lopez, Juan Carlos a; Miralles, Pilar a; Catalan, Pilar e; Resino, Salvador c,*

Journals@Ovid

Cytokine. 57(1):25-29, January 2012.

[Miscellaneous] [Short Communication]

AN: 00001705-201201000-00005.

<td colspan="">

Link to the Ovid Full Text or citation:

[Click here for full text options](http://ovidsp.ovid.com/ovidweb.cgi?T=JS&CSC=Y&NEWS=N&PAGE=fulltext&D=ovftm&AN=00001705-201201000-00005)

22.

The impact of liver disease aetiology and the stages of hepatic fibrosis on the performance of non-invasive fibrosis biomarkers: an international study of 2411 cases.

Sebastiani, G. 1; Castera, L. 2; Halfon, P. 3; Pol, S. 4; Mangia, A. 5; Di Marco, V. 6; Pirisi, M. 7; Voiculescu, M. 8; Bourliere, M. 9; Alberti, A. 1,10

Journals@Ovid

Alimentary Pharmacology & Therapeutics. 34(10):1202-1216, November 2011.

[Miscellaneous] [HEPATOLOGY]

AN: 00001716-201111010-00006.

<td colspan="">

Link to the Ovid Full Text or citation:

[Click here for full text options](http://ovidsp.ovid.com/ovidweb.cgi?T=JS&CSC=Y&NEWS=N&PAGE=fulltext&D=ovftm&AN=00001716-201111010-00006)

23.

Estimated Liver Fibrosis and its Impact on All-Cause Mortality of HCV-Monoinfected and HCV/HIV-Coinfected Drug Users.

Sanvisens, A. 1; Fuster, D. 1; Serra, I. 2; Tor, J. 1; Tural, C. 1; Rey-Joly, C. 1; Muga, R. 1

Journals@Ovid

Current HIV Research. 9(4):256-262, 2011.

[Article] [Articles]

AN: 00148464-201109040-00008.

<td colspan="">

Link to the Ovid Full Text or citation:

[Click here for full text options](http://ovidsp.ovid.com/ovidweb.cgi?T=JS&CSC=Y&NEWS=N&PAGE=fulltext&D=ovftm&AN=00148464-201109040-00008)

24.

Prospective Evaluation of FibroScan for the Diagnosis of Hepatic Fibrosis Compared with Liver Biopsy/AST Platelet Ratio Index and FIB-4 in Patients with Chronic HBV Infection.

Zhu, Xia; Wang, Li-Chun; Chen, En-Qiang; Chen, Xue-Bing; Chen, Li-Yu; Liu, Li; Lei, Xue-Zhong; Liu, Cong; Tang, Hong

Journals@Ovid

Digestive Diseases & Sciences. 56(9):2742-2749, September 2011.

[Article] [ORIGINAL ARTICLE]

AN: 00003455-201109000-00032.

<td colspan="">

Link to the Ovid Full Text or citation:

[Click here for full text options](http://ovidsp.ovid.com/ovidweb.cgi?T=JS&CSC=Y&NEWS=N&PAGE=fulltext&D=ovftm&AN=00003455-201109000-00032)

25.

Evaluation of Liver Fibrosis: Concordance Analysis between Noninvasive Scores (APRI and FIB-4) Evolution and Predictors in a Cohort of HIV-Infected Patients without Hepatitis C and B Infection.

Mendeni, Monia 1,a; Foca, Emanuele 1,a; Gotti, Daria 1; Ladisa, Nicoletta 3; Angarano, Gioacchino 3; Albini, Laura 1; Castelnuovo, Filippo 2; Carosi, Giampiero 1; Quiros-Roldan, Eugenia 1; Torti, Carlo 1

Journals@Ovid

Clinical Infectious Diseases. 52(9):1164-1173, May 01, 2011.

[Miscellaneous Article] [HIV/AIDS: Editor's Choice]

AN: 01451458-201105010-00019.

<td colspan="">

Link to the Ovid Full Text or citation:

[Click here for full text options](http://ovidsp.ovid.com/ovidweb.cgi?T=JS&CSC=Y&NEWS=N&PAGE=fulltext&D=ovftl&AN=01451458-201105010-00019)

26.

HIV Mono-infection Is Associated With FIB-4 - A Noninvasive Index of Liver Fibrosis - in Women.

Blackard, Jason T. 1; Welge, Jeffrey A. 2,3; Taylor, Lynn E. 4; Mayer, Kenneth H. 4; Klein, Robert S. 5; Celentano, David D. 6; Jamieson, Denise J. 7; Gardner, Lytt 8; Sherman, Kenneth E. 1

Journals@Ovid

Clinical Infectious Diseases. 52(5):674-680, March 1, 2011.

[Miscellaneous Article] [HIV/AIDS]

AN: 01451458-201103010-00027.

<td colspan="">

Link to the Ovid Full Text or citation:

[Click here for full text options](http://ovidsp.ovid.com/ovidweb.cgi?T=JS&CSC=Y&NEWS=N&PAGE=fulltext&D=ovftl&AN=01451458-201103010-00027)

27.

Non-Invasive Index for Predicting Significant Liver Fibrosis: Comparison of Diagnostic Performances in Patients with Chronic Hepatitis B and C.

Cheong, Jae Youn; Um, Soon Ho; Seo, Yeon Seok; Kim, Dong Joon; Hwang, Seong Gyu; Lee, Youn Jae; Cho, Mong; Yang, Jin Mo; Kim, Young Bae; Park, Young Nyun; Cho, Sung Won

Journals@Ovid

Digestive Diseases & Sciences. 56(2):555-563, February 2011.

[Article] [ORIGINAL ARTICLE]

AN: 00003455-201102000-00038.

<td colspan="">

Link to the Ovid Full Text or citation:

[Click here for full text options](http://ovidsp.ovid.com/ovidweb.cgi?T=JS&CSC=Y&NEWS=N&PAGE=fulltext&D=ovftl&AN=00003455-201102000-00038)

28.

Coinfection with hepatitis C virus, oxidative stress and antioxidant status in HIV-positive drug users in Miami *.

Baum, MK 1; Sales, S 1; Jayaweera, DT 2; Lai, S 3; Bradwin, G 4; Rafie, C 1; Page, JB 2; Campa, A 1

Journals@Ovid

HIV Medicine. 12(2):78-86, February 2011.

[Article] [Original research]

AN: 00133448-201102000-00002.

<td colspan="">

Link to the Ovid Full Text or citation:

[Click here for full text options](http://ovidsp.ovid.com/ovidweb.cgi?T=JS&CSC=Y&NEWS=N&PAGE=fulltext&D=ovftl&AN=00133448-201102000-00002)

29.

Assessment of liver fibrosis before and after antiviral therapy by different serum marker panels in patients with chronic hepatitis C.

Martinez, S. M. 1; Fernandez-Varo, G. 2; Gonzalez, P. 1; Sampson, E. 3; Bruguera, M. 1; Navasa, M. 1; Jimenez, W. 2; Sanchez-Tapias, J. M. 1; Forns, X. 1

Journals@Ovid

Alimentary Pharmacology & Therapeutics. 33(1):138-148, January 2011.

[Miscellaneous Article] [HEPATITISC]

AN: 00001716-201101000-00015.

<td colspan="">

Link to the Ovid Full Text or citation:

[Click here for full text options](http://ovidsp.ovid.com/ovidweb.cgi?T=JS&CSC=Y&NEWS=N&PAGE=fulltext&D=ovftl&AN=00001716-201101000-00015)

30.

Comparison of Surrogate Serum Markers and Transient Elastography (Fibroscan) for Assessing Cirrhosis in Patients with Chronic Viral Hepatitis.

Lee, Myoung Hee; Cheong, Jae Youn; Um, Soon Ho; Seo, Yeon Seok; Kim, Dong Joon; Hwang, Seong Gyu; Yang, Jin Mo; Han, Kwang-Hyub; Cho, Sung Won

Journals@Ovid

Digestive Diseases & Sciences. 55(12):3552-3560, December 2010.

[Article] [ORIGINAL ARTICLE]

AN: 00003455-201012000-00037.

<td colspan="">

Link to the Ovid Full Text or citation:

[Click here for full text options](http://ovidsp.ovid.com/ovidweb.cgi?T=JS&CSC=Y&NEWS=N&PAGE=fulltext&D=ovftl&AN=00003455-201012000-00037)

31.

Biomarkers of fibrosis and impaired liver function in chronic hepatitis C: how well do they predict clinical outcomes?.

Peters, Lars a; Rockstroh, Jurgen Kurt b

Journals@Ovid

Current Opinion in HIV & AIDS. 5(6):517-523, November 2010.

[Miscellaneous Article] [Biomarkers of outcomes of disease, treatment and complications: Edited by H. Clifford Lane and Jens D. Lundgren]

AN: 01222929-201011000-00011.

<td colspan="">

Link to the Ovid Full Text or citation:

[Click here for full text options](http://ovidsp.ovid.com/ovidweb.cgi?T=JS&CSC=Y&NEWS=N&PAGE=fulltext&D=ovftl&AN=01222929-201011000-00011)

32.

External Validation of P2/MS and Comparison with Other Simple Non-invasive Indices for Predicting Liver Fibrosis in HBV-Infected Patients.

Kim, Beom Kyung; Han, Kwang Hyub; Park, Jun Yong; Ahn, Sang Hoon; Chon, Chae Yoon; Kim, Ja Kyung; Paik, Yong Han; Lee, Kwan Sik; Park, Young Nyun; Kim, Do Young

Journals@Ovid

Digestive Diseases & Sciences. 55(9):2636-2643, September 2010.

[Article] [ORIGINAL ARTICLE]

AN: 00003455-201009000-00029.

<td colspan="">

Link to the Ovid Full Text or citation:

[Click here for full text options](http://ovidsp.ovid.com/ovidweb.cgi?T=JS&CSC=Y&NEWS=N&PAGE=fulltext&D=ovftl&AN=00003455-201009000-00029)

33.

Development of a non-invasive algorithm with transient elastography (Fibroscan) and serum test formula for advanced liver fibrosis in chronic hepatitis B.

WONG, G. L. H. *,+; WONG, V. W. S. *,+; CHOI, P. C. L. ++; CHAN, A. W. H. ++; CHAN, H. L. Y. *,+

Journals@Ovid

Alimentary Pharmacology & Therapeutics. 31(10):1095-1103, May 15, 2010.

[Article] [Original articles]

AN: 00001716-201005150-00005.

<td colspan="">

Link to the Ovid Full Text or citation:

[Click here for full text options](http://ovidsp.ovid.com/ovidweb.cgi?T=JS&CSC=Y&NEWS=N&PAGE=fulltext&D=ovftk&AN=00001716-201005150-00005)

34.

Assessment of Allograft Fibrosis by Transient Elastography and Noninvasive Biomarker Scoring Systems in Liver Transplant Patients.

Beckebaum, Susanne 1,2,5; Iacob, Speranta 2,3; Klein, Christian G. 1,2; Dechene, Alexander 2; Varghese, Joye 1; Baba, Hideo A. 4; Sotiropoulos, Georgios C. 1; Paul, Andreas 1; Gerken, Guido 2; Cicinnati, Vito R. 1,2

Journals@Ovid

Transplantation. 89(8):983-993, April 27, 2010.

[Miscellaneous Article] [Clinical and Translational Research]

AN: 00007890-201004270-00011.

<td colspan="">

Link to the Ovid Full Text or citation:

[Click here for full text options](http://ovidsp.ovid.com/ovidweb.cgi?T=JS&CSC=Y&NEWS=N&PAGE=fulltext&D=ovftk&AN=00007890-201004270-00011)

35.

Comparison of transient elastography and liver biopsy for the assessment of liver fibrosis in HIV/hepatitis C virus-coinfected patients and correlation with noninvasive serum markers.

Sanchez-Conde, M. 1; Montes-Ramirez, M. L. 2; Miralles, P. 1; Castro Alvarez, J. M. 2; Bellon, J. M. 1; Ramirez, M. 1; Arribas, J. R. 2; Gutierrez, I. 1; Lopez, J. C. 1; Cosin, J. 1; Alvarez, E. 1; Gonzalez, J. 2; Berenguer, J. 1

Journals@Ovid

Journal of Viral Hepatitis. 17(4):280-286, April 2010.

[Article] [ORIGINAL ARTICLES]

AN: 00043778-201004000-00008.

<td colspan="">

Link to the Ovid Full Text or citation:

[Click here for full text options](http://ovidsp.ovid.com/ovidweb.cgi?T=JS&CSC=Y&NEWS=N&PAGE=fulltext&D=ovftk&AN=00043778-201004000-00008)

36.

Validation of FIB-4 and comparison with other simple noninvasive indices for predicting liver fibrosis and cirrhosis in hepatitis B virus-infected patients.

Kim, Beom Kyung 1; Kim, Do Young 1,2,3; Park, Jun Yong 1,2,4; Ahn, Sang Hoon 1,2,4; Chon, Chae Yoon 1,2,4; Kim, Ja Kyung 1,2,4; Paik, Yong Han 1,2,4,5; Lee, Kwan Sik 1,2,4; Park, Young Nyun 3; Han, Kwang Hyub 1,2,4,5

Journals@Ovid

Liver International. 30(4):546-553, April 2010.

[Article] [Clinical Studies]

AN: 00137700-201004000-00009.

<td colspan="">

Link to the Ovid Full Text or citation:

[Click here for full text options](http://ovidsp.ovid.com/ovidweb.cgi?T=JS&CSC=Y&NEWS=N&PAGE=fulltext&D=ovftk&AN=00137700-201004000-00009)

37.

Diagnosis of Fibrosis and Cirrhosis Using Liver Stiffness Measurement in Nonalcoholic Fatty Liver Disease.

Wong, Vincent Wai-Sun 1,2; Vergniol, Julien 3; Wong, Grace Lai-Hung 1,2; Foucher, Juliette 3; Chan, Henry Lik-Yuen 1,2; Le Bail, Brigitte 4,5; Choi, Paul Cheung-Lung 6; Kowo, Mathurin 3; Chan, Anthony Wing-Hung 6; Merrouche, Wassil 3; Sung, Joseph Jao-Yiu 1,2; de Ledinghen, Victor 3,4,*

Journals@Ovid

Hepatology. 51(2):454-462, February 2010.

[Miscellaneous Article] [Steatohepatitis/Metabolic Liver Disease]

AN: 01515467-201002000-00016.

<td colspan="">

Link to the Ovid Full Text or citation:

[Click here for full text options](http://ovidsp.ovid.com/ovidweb.cgi?T=JS&CSC=Y&NEWS=N&PAGE=fulltext&D=ovftk&AN=01515467-201002000-00016)

38.

Diagnosis of advanced fibrosis in HIV and hepatitis C virus-coinfected patients via a new noninvasive index: the HGM-3 index.

Resino, S 1; Micheloud, D 1; Miralles, P 2; Bellon, J M 3; Vargas, A 1; Catalan, P 4; Alvarez, E 5; Cosin, J 2; Lorente, R 6; Lopez, J C 2; Munoz-Fernandez, M A 6; Berenguer, J 2

Journals@Ovid

HIV Medicine. 11(1):64-73, January 2010.

[Article] [Original research]

AN: 00133448-201001000-00006.

<td colspan="">

Link to the Ovid Full Text or citation:

[Click here for full text options](http://ovidsp.ovid.com/ovidweb.cgi?T=JS&CSC=Y&NEWS=N&PAGE=fulltext&D=ovftk&AN=00133448-201001000-00006)

39.

Complete blood count reflects the degree of oesophageal varices and liver fibrosis in virus-related chronic liver disease patients.

Lee, J.-H. 1,2; Yoon, J.-H. 1,2; Lee, C.-H. 1,3; Myung, S. J. 1,2; Keam, B. 1,4; Kim, B. H. 1,2; Chung, G. E. 1,2; Kim, W. 1,2; Kim, Y. J. 1,2; Jang, J. J. 5; Lee, H-S. 1,2

Journals@Ovid

Journal of Viral Hepatitis. 16(6):444-452, June 2009.

[Article] [ORIGINAL ARTICLES]

AN: 00043778-200906000-00008.

<td colspan="">

Link to the Ovid Full Text or citation:

[Click here for full text options](http://ovidsp.ovid.com/ovidweb.cgi?T=JS&CSC=Y&NEWS=N&PAGE=fulltext&D=ovftj&AN=00043778-200906000-00008)

40.

Saquinavir exposure in HIV-infected patients with chronic viral hepatitis.

Molto, Jose 1,*; Llibre, Josep Maria 2; Ribera, Esteban 3; Minguez, Carlos 4; del Rio, Jesus Sanchez 5; Pedrol, Enric 6; Vallecillo, Gabriel 7; Cedeno, Samandhy 8; Valle, Marta 9; Miranda, Cristina 1; Negredo, Eugenia 1; Clotet, Bonaventura 1,8; on behalf of the SQV-HEP Study Group +

Journals@Ovid

Journal of Antimicrobial Chemotherapy. 63(5):992-997, May 2009.

[Article] [Original research]

AN: 00004548-200905000-00022.

<td colspan="">

Link to the Ovid Full Text or citation:

[Click here for full text options](http://ovidsp.ovid.com/ovidweb.cgi?T=JS&CSC=Y&NEWS=N&PAGE=fulltext&D=ovftj&AN=00004548-200905000-00022)

41.

The accuracy of the FIB-4 index for the diagnosis of mild fibrosis in chronic hepatitis B.

MALLET, V. *,+,++,1; DHALLUIN-VENIER, V. *,+,1; ROUSSIN, C. [S]; BOURLIERE, M. [P]; PETTINELLI, M. E. **; GIRY, C. **; VALLET-PICHARD, A. *,+,++; FONTAINE, H. +,++; POL, S. *,+,++

Journals@Ovid

Alimentary Pharmacology & Therapeutics. 29(4):409-415, February 2009.

[Article] [Original article]

AN: 00001716-200902040-00006.

<td colspan="">

Link to the Ovid Full Text or citation:

[Click here for full text options](http://ovidsp.ovid.com/ovidweb.cgi?T=JS&CSC=Y&NEWS=N&PAGE=fulltext&D=ovftj&AN=00001716-200902040-00006)

42.

Evaluating the accuracy and increasing the reliable diagnosis rate of blood tests for liver fibrosis in chronic hepatitis C.

Cales, Paul 1; de Ledinghen, Victor 2; Halfon, Philippe 3; Bacq, Yannick 4; Leroy, Vincent 5; Boursier, Jerome 1; Foucher, Juliette 2; Bourliere, Marc 6; de Muret, Anne 7; Sturm, Nathalie 8; Hunault, Gilles 1; Oberti, Frederic 1

Journals@Ovid

Liver International. 28(10):1352-1362, November 2008.

[Article] [Clinical Studies]

AN: 00137700-200811000-00006.

<td colspan="">

Link to the Ovid Full Text or citation:

[Click here for full text options](http://ovidsp.ovid.com/ovidweb.cgi?T=JS&CSC=Y&NEWS=N&PAGE=fulltext&D=ovftj&AN=00137700-200811000-00006)

43.

Validation and Comparison of Simple Noninvasive Indexes for Predicting Liver Fibrosis in HIV-HCV-Coinfected Patients: ANRS CO3 Aquitaine Cohort.

Loko, Marc-Arthur M.D. 1; Castera, Laurent M.D., Ph.D. 2; Dabis, Francois M.D., Ph.D. 1,3; Le Bail, Brigitte M.D., Ph.D. 4; Winnock, Maria Ph.D. 1; Coureau, Gaelle M.D. 1; Bioulac-Sage, Paulette M.D., Ph.D. 4; de Ledinghen, Victor M.D., Ph.D. 2; Neau, Didier M.D., Ph.D. 1,5; the Groupe d'Epidemiologie Clinique du SIDA en Aquitaine (GECSA) 1,3

Journals@Ovid

American Journal of Gastroenterology. 103(8):1973-1980, August 2008.

[Editorial] [ORIGINAL CONTRIBUTION: LIVER AND BILIARY TRACT: Editorial]

AN: 00000434-200808000-00015.

<td colspan="">

Link to the Ovid Full Text or citation:

[Click here for full text options](http://ovidsp.ovid.com/ovidweb.cgi?T=JS&CSC=Y&NEWS=N&PAGE=fulltext&D=ovftj&AN=00000434-200808000-00015)

44.

Liver fibrosis on account of chronic hepatitis C is more severe in HIV-positive than HIV-negative patients despite antiretroviral therapy.

de Ledinghen, V. 1,2; Barreiro, P. 3; Foucher, J. 1; Labarga, P. 3; Castera, L. 1; Vispo, M. E. 3; Bernard, P.-H. 4; Martin-Carbonero, L. 3; Neau, D. 5; Garcia-Gasco, P. 3; Merrouche, W. 1; Soriano, V. 3

Journals@Ovid

Journal of Viral Hepatitis. 15(6):427-433, June 2008.

[Article] [ORIGINAL ARTICLE]

AN: 00043778-200806000-00004.

<td colspan="">

Link to the Ovid Full Text or citation:

[Click here for full text options](http://ovidsp.ovid.com/ovidweb.cgi?T=JS&CSC=Y&NEWS=N&PAGE=fulltext&D=ovftj&AN=00043778-200806000-00004)

45.

APRI, the FIB-4 score, and Forn's index have noninvasive diagnostic value for liver fibrosis in patients with chronic hepatitis B.

Ucar, Fatma a; Sezer, Sevilay d; Ginis, Zeynep a; Ozturk, Gulfer a; Albayrak, Aynur b; Basar, Omer c; Ekiz, Fuat c; Coban, Sahin c; Yuksel, Osman c; Armutcu, Ferah e; Akbal, Erdem f

Zhejiang University Full-Text Journals(LWW Journals)

European Journal of Gastroenterology & Hepatology. 25(9):1076-1081, September 2013.

[Article] [Original Articles: Hepatitis]

AN: 00042737-201309000-00010.

<td colspan="">

Link to the Ovid Full Text or citation:

[Click here for full text options](http://ovidsp.ovid.com/ovidweb.cgi?T=JS&CSC=Y&NEWS=N&PAGE=fulltext&D=yrovfto&AN=00042737-201309000-00010)

46.

Evaluation of seven noninvasive models in staging liver fibrosis in patients with chronic hepatitis B virus infection.

Ma, Jing; Jiang, Yongfang; Gong, Guozhong

Zhejiang University Full-Text Journals(LWW Journals)

European Journal of Gastroenterology & Hepatology. 25(4):428-434, April 2013.

[Article] [Original Articles: Hepatitis]

AN: 00042737-201304000-00007.

<td colspan="">

Link to the Ovid Full Text or citation:

[Click here for full text options](http://ovidsp.ovid.com/ovidweb.cgi?T=JS&CSC=Y&NEWS=N&PAGE=fulltext&D=yrovftn&AN=00042737-201304000-00007)

47.

The Accuracy of Noninvasive Methods in Predicting the Development of Hepatocellular Carcinoma and Hepatic Decompensation in Patients With Chronic Hepatitis B.

Chon, Young Eun MD *,+,++; Jung, Eun Suk MD *,+,++; Park, Jun Yong MD *,+,++,[S]; Kim, Do Young MD, PhD *,+,++,[S]; Ahn, Sang Hoon MD, PhD *,+,++,[S]; Han, Kwang-Hyub MD, PhD *,+,++,[S]; Chon, Chae Yoon MD, PhD *,+,++,[S]; Jung, Kyu Sik MD *,+,++; Kim, Seung Up MD *,+,++,[S]

Zhejiang University Full-Text Journals(LWW Journals)

Journal of Clinical Gastroenterology. 46(6):518-525, July 2012.

[Article] [LIVER, PANCREAS AND BILIARY TRACT: Original Article]

AN: 00004836-201207000-00016.

<td colspan="">

Link to the Ovid Full Text or citation:

[Click here for full text options](http://ovidsp.ovid.com/ovidweb.cgi?T=JS&CSC=Y&NEWS=N&PAGE=fulltext&D=yrovftn&AN=00004836-201207000-00016)

48.

Assessing mortality in women with hepatitis C virus and HIV using indirect markers of fibrosis.

Bambha, Kiran a; Pierce, Christopher b; Cox, Christopher b; French, Audrey L. c; Tien, Phyllis C. a,i; Sharp, Gerald B. d; Augenbraun, Michael e; Glesby, Marshall J. f; Villacres, Maria C. g; Plankey, Michael h; Strickler, Howard D. j; Gange, Stephen J. b; Peters, Marion G. a

Zhejiang University Full-Text Journals(LWW Journals)

AIDS. 26(5):599-607, March 13, 2012.

[Article] [CLINICAL SCIENCE]

AN: 00002030-201203130-00009.

<td colspan="">

Link to the Ovid Full Text or citation:

[Click here for full text options](http://ovidsp.ovid.com/ovidweb.cgi?T=JS&CSC=Y&NEWS=N&PAGE=fulltext&D=yrovftm&AN=00002030-201203130-00009)

49.

Biomarkers of fibrosis and impaired liver function in chronic hepatitis C: how well do they predict clinical outcomes?.

Peters, Lars a; Rockstroh, Jurgen Kurt b

Zhejiang University Full-Text Journals(LWW Journals)

Current Opinion in HIV & AIDS. 5(6):517-523, November 2010.

[Miscellaneous Article] [Biomarkers of outcomes of disease, treatment and complications: Edited by H. Clifford Lane and Jens D. Lundgren]

AN: 01222929-201011000-00011.

<td colspan="">

Link to the Ovid Full Text or citation:

[Click here for full text options](http://ovidsp.ovid.com/ovidweb.cgi?T=JS&CSC=Y&NEWS=N&PAGE=fulltext&D=yrovftl&AN=01222929-201011000-00011)

50.

Assessment of Allograft Fibrosis by Transient Elastography and Noninvasive Biomarker Scoring Systems in Liver Transplant Patients.

Beckebaum, Susanne 1,2,5; Iacob, Speranta 2,3; Klein, Christian G. 1,2; Dechene, Alexander 2; Varghese, Joye 1; Baba, Hideo A. 4; Sotiropoulos, Georgios C. 1; Paul, Andreas 1; Gerken, Guido 2; Cicinnati, Vito R. 1,2

Zhejiang University Full-Text Journals(LWW Journals)

Transplantation. 89(8):983-993, April 27, 2010.

[Miscellaneous Article] [Clinical and Translational Research]

AN: 00007890-201004270-00011.

<td colspan="">

Link to the Ovid Full Text or citation:

[Click here for full text options](http://ovidsp.ovid.com/ovidweb.cgi?T=JS&CSC=Y&NEWS=N&PAGE=fulltext&D=yrovftk&AN=00007890-201004270-00011)

51.

Prospective Evaluation of FibroScan for the Diagnosis of Hepatic Fibrosis Compared with Liver Biopsy/AST Platelet Ratio Index and FIB-4 in Patients with Chronic HBV Infection

Zhu, Xia [Author]; Wang, Li-Chun [Author]; Chen, En-Qiang [Author]; Chen, Xue-Bing [Author]; Chen, Li-Yu [Author]; Liu, Li [Author]; Lei, Xue-Zhong [Author]; Liu, Cong [Author]; Tang, Hong [Author, Reprint Author; E-mail: htang6198@hotmail.com].

BIOSIS Previews

Digestive Diseases & Sciences. 56(9). SEP 2011. 2742-2749.

[Article]

AN: PREV201100633892

Year of Publication

2011

<td colspan="">

Link to the Ovid Full Text or citation:

[Click here for full text options](http://ovidsp.ovid.com/ovidweb.cgi?T=JS&CSC=Y&NEWS=N&PAGE=fulltext&D=biop37&AN=PREV201100633892)

52.

A New Model Using Routinely Available Clinical Parameters to Predict Significant Liver Fibrosis in Chronic Hepatitis B

Seto, Wai-Kay [Author, Reprint Author]; Lee, Chun-Fan [Author]; Lai, Ching-Lung [Author]; Ip, Philip P. C. [Author]; Fong, Daniel Yee-Tak [Author]; Fung, James [Author]; Wong, Danny Ka-Ho [Author]; Yuen, Man-Fung [Author; E-mail: mfyuen@hkucc.hku.hk].

BIOSIS Previews

PLoS One. 6(8). AUG 11 2011. Article No.: e23077.

[Article]

AN: PREV201100611898

Year of Publication

2011

<td colspan="">

Link to the Ovid Full Text or citation:

[Click here for full text options](http://ovidsp.ovid.com/ovidweb.cgi?T=JS&CSC=Y&NEWS=N&PAGE=fulltext&D=biop37&AN=PREV201100611898)

53.

Comparison of non-invasive assessment to diagnose liver fibrosis in chronic hepatitis B and C patients

Stibbe, Krista J. M. [Author]; Verveer, Claudia [Author]; Francke, Jan [Author]; Hansen, Bettina E. [Author]; Zondervan, Pieter E. [Author]; Kuipers, Ernst J. [Author]; de Knegt, Robert J. [Author]; van Vuuren, Anneke J. [Author, Reprint Author; E-mail: a.vanvuuren@erasmusmc.nl].

BIOSIS Previews

Scandinavian Journal of Gastroenterology. 46(7-8). JUL 2011. 962-972.

[Article]

AN: PREV201100512463

Year of Publication

2011

<td colspan="">

Link to the Ovid Full Text or citation:

[Click here for full text options](http://ovidsp.ovid.com/ovidweb.cgi?T=JS&CSC=Y&NEWS=N&PAGE=fulltext&D=biop37&AN=PREV201100512463)

54.

Hepatitis B Virus Genotype G: Prevalence and Impact in Patients Co-Infected With Human Immunodeficiency Virus

Dao, Doan Y. [Author]; Balko, Jody [Author]; Attar, Nahid [Author]; Neak, Enayet [Author]; Yuan, He-Jun [Author]; Lee, William M. [Author]; Jain, Mamta K. [Author, Reprint Author; E-mail: mamta.jain@utsouthwestern.edu].

BIOSIS Previews

Hepatitis B virus S gene [Hepadnaviridae] Journal of Medical Virology. 83(9). SEP 2011. 1551-1558.

[Article]

AN: PREV201100507800

Year of Publication

2011

<td colspan="">

Link to the Ovid Full Text or citation:

[Click here for full text options](http://ovidsp.ovid.com/ovidweb.cgi?T=JS&CSC=Y&NEWS=N&PAGE=fulltext&D=biop37&AN=PREV201100507800)

55.

HIV Mono-infection Is Associated With FIB-4-A Noninvasive Index of Liver Fibrosis - in Women

Blackard, Jason T. [Author, Reprint Author; E-mail: jason.blackard@uc.edu]; Welge, Jeffrey A. [Author]; Taylor, Lynn E. [Author]; Mayer, Kenneth H. [Author]; Klein, Robert S. [Author]; Celentano, David D. [Author]; Jamieson, Denise J. [Author]; Gardner, Lytt [Author]; Sherman, Kenneth E. [Author].

BIOSIS Previews

Clinical Infectious Diseases. 52(5). MAR 1 2011. 674-680.

[Article]

AN: PREV201100179305

Year of Publication

2011

<td colspan="">

Link to the Ovid Full Text or citation:

[Click here for full text options](http://ovidsp.ovid.com/ovidweb.cgi?T=JS&CSC=Y&NEWS=N&PAGE=fulltext&D=biop37&AN=PREV201100179305)

56.

Development of a non-invasive algorithm with transient elastography (Fibroscan) and serum test formula for advanced liver fibrosis in chronic hepatitis B

Wong, G. L. H. [Author]; Wong, V. W. S. [Author]; Choi, P. C. L. [Author]; Chan, A. W. H. [Author]; Chan, H. L. Y. [Author, Reprint Author; E-mail: hlychan@cuhk.edu.hk].

BIOSIS Previews

Alimentary Pharmacology & Therapeutics. 31(10). MAY 15 2010. 1095-1103.

[Article]

AN: PREV201000252538

Year of Publication

2010

<td colspan="">

Link to the Ovid Full Text or citation:

[Click here for full text options](http://ovidsp.ovid.com/ovidweb.cgi?T=JS&CSC=Y&NEWS=N&PAGE=fulltext&D=biop36&AN=PREV201000252538)

57.

External Validation of P2/MS and Comparison with Other Simple Non-invasive Indices for Predicting Liver Fibrosis in HBV-Infected Patients

Kim, Beom Kyung [Author]; Han, Kwang Hyub [Author]; Park, Jun Yong [Author]; Ahn, Sang Hoon [Author]; Chon, Chae Yoon [Author]; Kim, Ja Kyung [Author]; Paik, Yong Han [Author]; Lee, Kwan Sik [Author]; Park, Young Nyun [Author]; Kim, Do Young [Author, Reprint Author; E-mail: dyk1025@yuhs.ac].

BIOSIS Previews

Digestive Diseases & Sciences. 55(9). SEP 2010. 2636-2643.

[Article]

AN: PREV201000488931

Year of Publication

2010

<td colspan="">

Link to the Ovid Full Text or citation:

[Click here for full text options](http://ovidsp.ovid.com/ovidweb.cgi?T=JS&CSC=Y&NEWS=N&PAGE=fulltext&D=biop36&AN=PREV201000488931)

58.

Comparison of Elastography, Serum Marker Scores, and Histology for the Assessment of Liver Fibrosis in Hepatitis B Virus (HBV)-Infected Patients in Burkina Faso

Bonnard, Philippe [Author, Reprint Author; E-mail: philippe.bonnard@tnn.aphp.fr]; Sombie, Roger [Author; E-mail: docsombf@yahoo.fr]; Lescure, Francois-Xavier [Author; E-mail: xavier.lescure@tnn.aphp.fr]; Bougouma, Alain [Author; E-mail: alain.bougouma@sante.gov.bf]; Guiard-Schmid, Jean Baptiste [Author; E-mail: guiardschmidjb@bf.afro.who.int]; Poynard, Thierry [Author; E-mail: tpoynard@teaser.fr]; Cales, Paul [Author; E-mail: paul.cales@univ-angers.fr]; Housset, Chantal [Author; E-mail: chantal.housset@tnn.aphp.fr]; Callard, Patrice [Author; E-mail: patrice.callard@tnn.aphp.fr]; Le Pendeven, Catherine [Author; E-mail: catherine.le-pendeven@tnn.aphp.fr]; Drabo, Joseph [Author; E-mail: j_drabo@hotmail.com]; Carrat, Fabrice [Author; E-mail: fcarrat@u707.jussieu.fr]; Pialoux, Gilles [Author; E-mail: gilles.pialoux@tnn.aphp.fr].

BIOSIS Previews

American Journal of Tropical Medicine & Hygiene. 82(3). MAR 2010. 454-458.

[Article]

AN: PREV201000202058

Year of Publication

2010

<td colspan="">

Link to the Ovid Full Text or citation:

[Click here for full text options](http://ovidsp.ovid.com/ovidweb.cgi?T=JS&CSC=Y&NEWS=N&PAGE=fulltext&D=biop36&AN=PREV201000202058)

59.

Validation of FIB-4 and comparison with other simple noninvasive indices for predicting liver fibrosis and cirrhosis in hepatitis B virus-infected patients

Kim, Beom Kyung [Author]; Kim, Do Young [Author]; Park, Jun Yong [Author]; Ahn, Sang Hoon [Author]; Chon, Chae Yoon [Author]; Kim, Ja Kyung [Author]; Paik, Yong Han [Author]; Lee, Kwan Sik [Author]; Park, Young Nyun [Author]; Han, Kwang Hyub [Author, Reprint Author; E-mail: gihankhys@yuhs.ac].

BIOSIS Previews

Liver International. 30(4). APR 2010. 546-553.

[Article]

AN: PREV201000185476

Year of Publication

2010

<td colspan="">

Link to the Ovid Full Text or citation:

[Click here for full text options](http://ovidsp.ovid.com/ovidweb.cgi?T=JS&CSC=Y&NEWS=N&PAGE=fulltext&D=biop36&AN=PREV201000185476)

60.

Saquinavir exposure in HIV-infected patients with chronic viral hepatitis

Molto, Jose [Author, Reprint Author; E-mail: jmolto@flsida.org]; Maria Llibre, Josep [Author]; Ribera, Esteban [Author]; Minguez, Carlos [Author]; Sanchez del Rio, Jesus [Author]; Pedrol, Enric [Author]; Vallecillo, Gabriel [Author]; Cedeno, Samandhy [Author]; Valle, Marta [Author]; Miranda, Cristina [Author]; Negredo, Eugenia [Author]; Clotet, Bonaventura [Author]; SQV-HEP Study Grp [Author].

BIOSIS Previews

Journal of Antimicrobial Chemotherapy. 63(5). MAY 2009. 992-997.

[Article]

AN: PREV200900292074

Year of Publication

2009

<td colspan="">

Link to the Ovid Full Text or citation:

[Click here for full text options](http://ovidsp.ovid.com/ovidweb.cgi?T=JS&CSC=Y&NEWS=N&PAGE=fulltext&D=biop35&AN=PREV200900292074)

61.

The accuracy of the FIB-4 index for the diagnosis of mild fibrosis in chronic hepatitis B

Mallet, V. [Author, Reprint Author; E-mail: vincent.mallet@cch.aphp.fr]; Dhalluin-Venier, V. [Author]; Roussin, C. [Author]; Bourliere, M. [Author]; Pettinelli, M. E. [Author]; Giry, C. [Author]; Vallet-Pichard, A. [Author]; Fontaine, H. [Author]; Pol, S. [Author].

BIOSIS Previews

Alimentary Pharmacology & Therapeutics. 29(4). FEB 15 2009. 409-415.

[Article]

AN: PREV200900104972

Year of Publication

2009

<td colspan="">

Link to the Ovid Full Text or citation:

[Click here for full text options](http://ovidsp.ovid.com/ovidweb.cgi?T=JS&CSC=Y&NEWS=N&PAGE=fulltext&D=biop35&AN=PREV200900104972)

62.

APRI and FIB-4 Scores Are Useful After Liver Transplantation Independently of Etiology

Pissaia, A. Jr. [Author]; Borderie, D. [Author]; Bernard, D. [Author]; Scatton, O. [Author]; Calmus, Y. [Author]; Conti, F. [Author, Reprint Author; E-mail: filomena.conti@cch.aphp.fr].

BIOSIS Previews

Transplantation Proceedings. 41(2). MAR 2009. 679-681.

[Article]

AN: PREV200900286053

Year of Publication

2009

<td colspan="">

Link to the Ovid Full Text or citation:

[Click here for full text options](http://ovidsp.ovid.com/ovidweb.cgi?T=JS&CSC=Y&NEWS=N&PAGE=fulltext&D=biop35&AN=PREV200900286053)

63.

Predictive value for low level hepatic fibrosis in chronic hepatitis C (cHC) patients: Comparison of five non-invasive indices

Yu, Ira [Author]; Dreizen, David [Author]; Talal, Andrew [Author]; Gish, Robert G. [Author]; Bonacini, Maurizio [Author].

BIOSIS Previews

Gastroenterology. 132(4, Suppl. 2). APR 2007. A786.

[Meeting]

AN: PREV200700606164

Year of Publication

2007

<td colspan="">

Link to the Ovid Full Text or citation:

[Click here for full text options](http://ovidsp.ovid.com/ovidweb.cgi?T=JS&CSC=Y&NEWS=N&PAGE=fulltext&D=biop33&AN=PREV200700606164)
